# Transcriptomics reveals the molecular processes of light-induced rapid darkening of the non-obligate cave dweller *Oreolalax rhodostigmatus* (Megophryidae, Anura) and their genetic basis of pigmentation strategy

**DOI:** 10.1186/s12864-018-4790-y

**Published:** 2018-05-31

**Authors:** Wei Zhu, Lusha Liu, Xungang Wang, Xinyu Gao, Jianping Jiang, Bin Wang

**Affiliations:** 1 CAS Key Laboratory of Mountain Ecological Restoration and Bioresource Utilization & Ecological Restoration Biodiversity Conservation Key Laboratory of Sichuan Province, Chengdu Institute of Biology, Chinese Academy of Sciences, Chengdu, 610041 China; 2 University of Chinese Academy of Sciences, Beijing, 100049 China

**Keywords:** Cave dweller, Melanocortin 1 receptor, Pigment regression, Melanogenesis, Morphological color change

## Abstract

**Background:**

Vertebrates use different pigmentation strategies to adapt to various environments. A large amount of research has been done on disclosing the mechanisms of pigmentation strategies in vertebrates either under light, or, living in constant darkness. However, less attention has been paid to non-obligate, darkness dwellers. Red-spotted toothed toads *Oreolalax rhodostigmatus* (Megophryidae; Anura) from the karst mountainous region of southwestern China are non-obligate cave dwellers. Most tadpoles of the species possess transparent skin as they inhabit the dark karst caves. But remarkably, the transparent tadpoles can darken just within 15 h once exposed to light. Obviously, it is very significant to reveal molecular mechanisms of the unexpected rapid-darkening phenomenon.

**Results:**

We compared the transcriptomes of *O. rhodostigmatus* tadpoles with different durations of light exposure to investigate the cellular processes and potential regulation signals for their light-induced rapid darkening. Genes involved in melanogenesis (i.e. TYR, TYRP1 and DCT) and melanocyte proliferation, as well as their transcriptional factor (MITF), showed light-induced transcription, suggesting a dominating role of morphological color change (MCC) in this process. Transcription of genes related to growth factor, MAPK and PI3K-Akt pathways increased with time of light exposure, suggesting that light could induce significant growth signal, which might facilitate the rapid skin darkening. Most importantly, an in-frame deletion of four residues was identified in *O. rhodostigmatus* melanocortin-1 receptor (MC1R), a critical receptor in MCC. This deletion results in a more negatively charged ligand pocket with three stereo-tandem aspartate residues. Such structural changes likely decrease the constitutive activity of MC1R, but increase its ligands-dependent activity, thus coordinating pigment regression and rapid melanogenesis in the dark and light, respectively.

**Conclusion:**

Our study suggested that rapid MCC was responsible for the light-induced rapid darkening of *O. rhodostigmatus* tadpoles. Genetic mutations of MC1R in them could explain how these non-obligate cave dwellers coordinate pigment regression and robust melanogenesis in darkness and light, respectively. To our knowledge, this is the first study that reports the association between pigmentation phenotype adaptation and MC1R mutations in amphibians and/or in non-obligate cave dwellers.

**Electronic supplementary material:**

The online version of this article (10.1186/s12864-018-4790-y) contains supplementary material, which is available to authorized users.

## Background

Vertebrates use different pigmentation strategies to adapt to various environments [1, 2]. For species living in environments with diurnal and seasonal changing light, skin pigment is necessary for preventing optical damage, and pigmentation configurations matching to the background are beneficial for their survival [3–6]. On the contrary, for species living in constant darkness (i.e. inside the caves), pigment regression is a common survival strategy [7–12], as pigmentation can be resource-consuming [13]. In these animals, the capacity to generate pigmentation is completely or partly lost [8–12]. Beside these two well-studied categories, there is the third category of animals, who may experience both dark and light conditions in their life histories (i.e. non-obligate cave dwellers). However, their pigmentation strategy and mechanisms are less concerned.

**Fig. 1:**
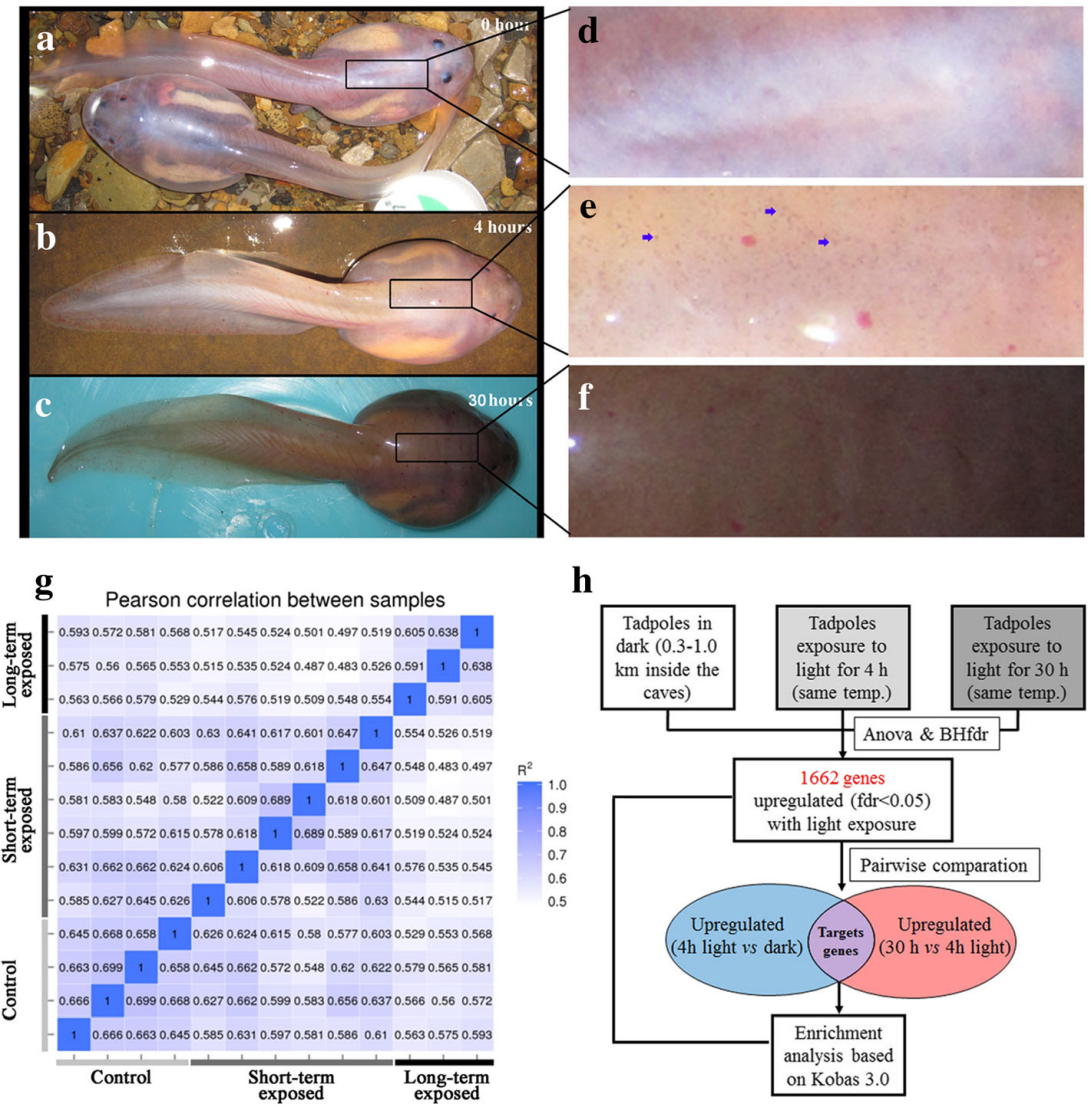
Phenotypes of *O. rhodostigmatus* tadpoles and transcriptomes analyses flow. **a**-**c**, Phenotypes of *O. rhodostigmatus* tadpoles in control group, short-term exposed group and long-term exposed group. **d**-**f**, Dorsal skin of *O. rhodostigmatus* tadpoles, blue arrows indicate typical black spots in short-term exposed larvae. **g**, Analyses flow of transcriptome data. **h**, Correlations of genes expression pattern between samples. A higher squared correlation coefficient (R^2^, 0–1) indicates more similar expression profiles between samples

Red-spotted toothed toad species *Oreolalax rhodostigmatus* (Megophryidae, Anura) is a typical non-obligate cave dweller, which is distributed in the karst mountainous region (altitude 500–2400 m. a. s. l.) of southwestern China [14]. Most tadpole populations of the species generally live in dark caves for several years with transparent skin as pigment regression, but meanwhile, some tadpole populations could also inhabit the “out-side and light” streams and possess black brown body color similar to their juvenile and adult frogs usually foraging outside the caves and generally possessing dark black body color [15, 16]. The most noticeable thing is that the transparent *O. rhodostigmatus* tadpoles could undergo fast and drastic darkening once exposed to the sunlight, with vast array of black spots presented within 4 h and whole-body brown to black skin color within 15 h (Fig. 1 a-f). The remarkable pigmentation capacity of the species indicates that their body probably has an “optical switch” for pigmentation to coordinate the opposite pigmentation requirements in darkness and light. Nevertheless, to present, there is no work focusing on disclosing the mechanisms of this fascinating phenomena.

Generally, light can induce physiological color change (PCC) and morphological color change (MCC) in animals. PCC is defined as rapid color change (within several hours) facilitated by the dispersion of pigment granules (color deepening) and aggregation (color fading) within chromatophores without pigment synthesis [17–19]. It is commonly observed in the color change of fish, amphibians and reptiles. PCC is mainly regulated by hormones, whose release is responsive to light [20]. Hormones inducing pigment dispersion in frogs includes alpha-melanocyte-stimulating hormone (α-MSH), adrenocorticotropic hormone (ACTH) and melanin-concentrating hormone (MCH), while those induce pigment aggregation include melatonin and adrenaline [1]. α-MSH is the most studied hormone involved in PCC. After incised from its precursor proopiomelanocortin protein (POMC), α-MSH binds to its receptor, melanocortin-1 receptor (MC1R), expressed on the cell membrane of chromatophores to stimulate pigment dispersion [17]. All the three major chromatophore types (melanophore, red erythrophore and yellow xanthophore) share similar PCC regulation patterns and mechanisms in frogs [1]. MCC is characterized by pigment synthesis, chromatophore proliferation and chromatophore. This kind of color changes is relatively slow, generally occurring over days or weeks [2, 6, 21]. The molecular processes of MCC have been well studied in melanocytes in mammals. Melanin synthesis in melanocytes, called melanogenesis, also requires the binding of α-MSH to MC1R, which activate the transcription of microphthalmia-associated transcription factor (MITF), the key transcriptional factor in melanocyte differentiation and melanogenesis [22, 23]. Meanwhile, activation of MAPK and PI3K-Akt pathways, which activated by growth factors released from keratinocyte in response to ultraviolet radiation, is responsible for phosphorylating MITF protein to its activation form [24–26]. Then, MITF activate melanocyte differentiation and expression of tyrosinase (TYR), tyrosinase-related protein 1 (TYRP1) and dopachrome tautomerase (DCT) for melanin synthesis. The molecular processes of MCC in frogs are likely similar to that of mammals, as α-MSH, receptor tyrosine kinase and MITF has been reported for early development of chromatophores in fishes and frogs [26–33].

In gene levels, the functional mutations in genes responsible for regulation of pigmentation are widely identified in vertebrates. For example, defects in genes involved in melanin biosynthesis (i.e., OCA2 and TYR) were always identified in cave fishes [8, 11, 12, 34]. Despite the large number of potential targets, only a handful of genes have been identified to contribute to genetic color adaptation in many animal taxa. Of these, MC1R are among the most widely studied pigmentation genes in mammals, birds, reptiles and fish [35], and some studies now show a link between variation in MC1R and pigmentation in numerous vertebrates [35–39]. MC1R is a member of the G protein-coupled receptor superfamily, consisted of 7 transmembrane fragments, 3 extracellular loops, 3 intracellular loops, 1 N-terminus and 1 C-terminus. Known mutations are largely interspersed throughout the transmembrane fragments and loops. The membrane/extracellular junctions of the second and third transmembrane domains (M/EJTD), which are negatively charged, is likely the site of electrostatic interaction with the arginine residue in α-MSH [40]. Mutations introducing basic residues or eliminating acid residues in this region always result in enhanced constitutive activity of MC1R in manner of ligand mimic [41], but also reduced agonist binding activity [40]. Interestingly, though melanistic phenotypes in a certain species are always associated with basic residues introducing and/or acid residues eliminating in these regions [41–50], pigment regression or pale phenotypes have not been identified to be granted by mutations acidifying this region [9, 51, 52]. As note, there is no report in amphibians in regard to the associations between color adaptation and variations of MC1R genes.

Hence, in this study, we try to uncover the mechanisms of the remarkable rapid darkening in *O. rhodostigmatus* tadpoles using comparative transcriptomics. Firstly, the coloration type was determined by analyzing the changes of expression pattern of genes involved in pigmentation with light exposure duration. Secondly, major light-induced transcriptional events were highlighted with gene differential expression and functional enrichment analyses, and their potential contributions to light-induced rapid darkening in *O. rhodostigmatus* were discussed. Thirdly, genes potentially contributing to genetic adaptation of coloration were analyzed by sequence alignments and protein homologous modeling to screen functional evolution responsible for the pigmentation strategy of *O. rhodostigmatus*.

## Results

### Summary of transcriptome assembly and transcript annotation

A total of 249,088 unigenes were obtained from 13 cDNA libraries, with their sequencing quality summarized in Additional file 1: Table S1. The mean length and N50 of unigenes were 1148 bp and 1928 bp, respectively (see Additional file 2: Figure S1 for length distribution). In total, 76,223, 57,186, 51,598, 81,733, 77,971, 81,074 and 36,301 unigenes were annotated in NR, NT, KO, SwissProt, PFAM, GO and KOG data bases, respectively (see Additional file 3: Table S2 for annotation details). Their expression levels (presented as FPKM) were summarized in Additional file 4: Table S3. Overall, the intra-group correlations are higher than inter-group ones in this study (Fig. 1g), supporting the validity of our transcriptome data.

A total of 1662 light inducible genes were identified (fdr < 0.05, one-way ANOVA). Among these, 213 ones show upregulation in subsequent pairwise comparisons: “short-term exposed *vs* control” and “long-term exposed *vs* short-term exposed” (Fig. 1h). These two groups of genes were respectively queried against KEGG database for enrichment analysis, and they shared most significantly enriched pathways (see Additional file 5: Table S4 and Additional file 6: Table S5 for details), suggesting that the results of enrichment analyses were not sensitive to the thresholds of differentially expressed genes.

### Coloration type of light-induced darkening

**Fig. 2:**
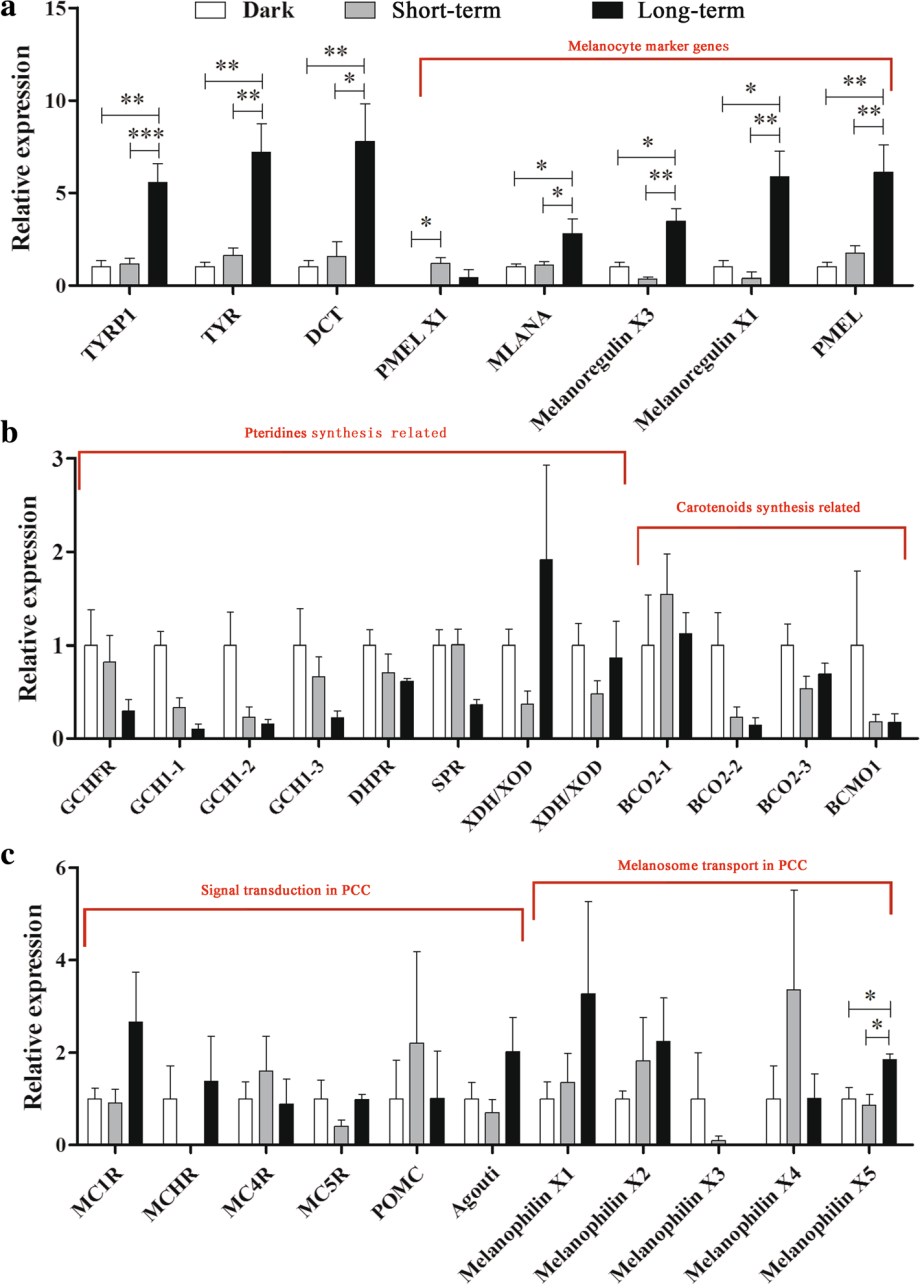
Transcriptional variation of coloration related genes in response to light exposure. **a**, Melanogenesis effector genes and melanocyte marker genes. **b**, Genes involved in synthesis of carotenoids and pteridines. **c**, Genes involved in PCC. Each column represents a mean ± SE, ***: *p* < 0.001, **: *p* < 0.01, *: *p* < 0.05 (T-test). TYRP1, tyrosinase-related protein 1; TYR, tyrosinase; DCT, dopachrome tautomerase; MLANA, melanoma antigen recognized by T-cells; Melanoregulin X1, melanoregulin isoform X1; Melanoregulin X3, melanoregulin isoform X3; PMEL, premelanosome protein precursor; PMEL X1, melanocyte protein PMEL isoform X1; DHPR, dihydropteridine reductase; GCHFR, GTP cyclohydrolase 1 feedback regulatory protein; GCH1, GTP cyclohydrolase 1; SPR, sepiapterin reductase; XDH/XOD, xanthine dehydrogenase/oxidase; BCO2, beta,beta-carotene 9′,10′-oxygenase; BCMO1, beta,beta-carotene 15,15′-monooxygenase. MC1R, melanocyte-stimulating hormone receptor / melanocortin receptor 1; MCHR, melanin-concentrating hormone receptor; Agouti, agouti signaling peptide; MC4R, melanocortin receptor 4; MC5R, melanocortin receptor 5; POMC, pro-opiomelanocortin

**Fig. 3:**
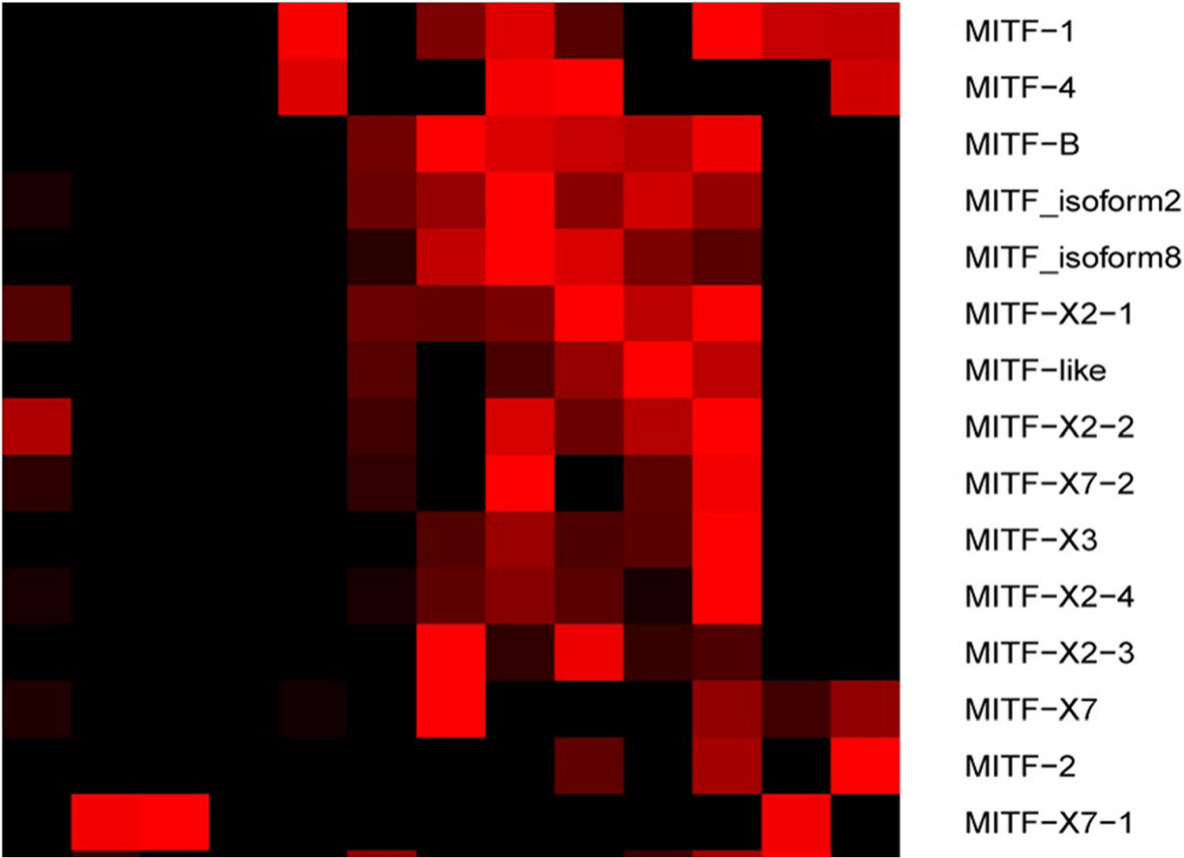
Expression heat map of MITF transcripts, expression level was scaled to 0–1

Transcription of effector genes in melanogenesis (i.e., TYR, TYRP1 and DCT) and marker genes of melanocyte (PMEL isoform X1, melanoma antigen recognized by T-cells 1 isoform X1/MELANA, Melanoregulin X1, Melanoregulin X3, and premelanosome protein precursor/PMEL) were increased with light exposure in *O. rhodostigmatus* tadpoles (Fig. 2a). Their transcriptional factor, MITF, was also transcriptionally activated by light exposure, with most of its transcripts peaked after short-term light exposure (Fig. 3). Xanthophores and erythrophores show no sign of response to light in manner of MCC due to genes involved in synthesis or metabolism of carotenoids (beta, beta-carotene 9′,10′-oxygenase isoform X1/BCO2 and beta, beta-carotene 15,15′-monooxygenase/BCMO1) and pteridines (dihydropteridine reductase/DHPR, GTP cyclohydrolase 1 feedback regulatory protein/GCHFR, GTP cyclohydrolase 1/GCH1, sepiapterin reductase/SPR and xanthine dehydrogenase/oxidase/XDH/XOD) were not upregulated, or even downregulated (Fig. 2b). In addition, neither MC1R, MC4R, MC5R, MCH receptor (MCHR), PMOC and agouti signal peptide (ASIP, antagonist of MC1R), which participate in signal transduction of pigment dispersion/aggregation, nor melanophilin proteins (excepting melanophilin 5), which were responsible for transport of melanosome, were transcriptionally upregulated by light (Fig. 2c).

### Light-induced major transcriptional events

**Fig. 4:**
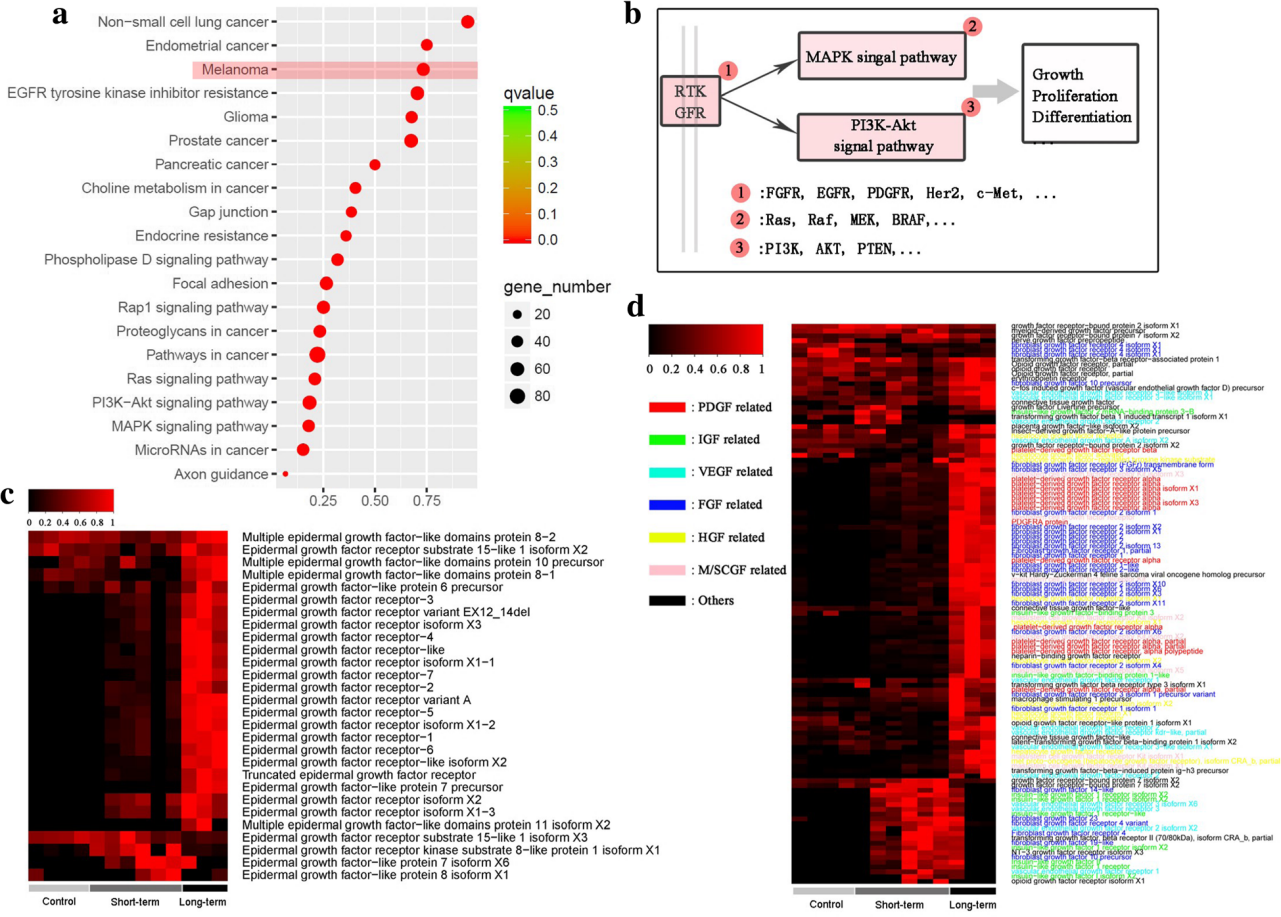
Primary transcriptional variations in response to light exposure in *O. rhodostigmatus* tadpoles. **a**, Top 20 (sorted by *q* value) enriched pathways/processes based on core light inducible gene. Rich factor is the ratio between number of gene enriched in a pathway and the total number of genes in this pathway. **b**, The consensus signal transduction unit among enriched pathways/processes. **c**, Expression heat map of genes related to epidermal growth factor, expression level was scaled to 0–1. **d**, Expression heat map of genes related to other growth factors, expression level was scaled to 0–1

Here, we focused on the results of enrichment analysis based on the 213 light-inducible genes. The top 20 (sorted by *q* value) enriched pathways/processes mainly referred to cancer and signal transduction (Fig. 4a). Genes enriched in these pathways/processes were further compared and integrated into a consensus module manually. This module was composed of growth factor receptors (GFRs), core components of MAPK signal pathway, and core components of PI3K-Akt signal pathway (Fig. 4b). In addition, growth factor-related genes constituted the largest gene group among light inducible genes. Most growth factor-related genes identified in our study are transcriptionally responsive to light exposure (Fig. 4c & d). Those related to epidermal growth factor (EGF), platelet-derived growth factor (PDGF), fibroblast growth factor (FGF), hepatocyte growth factor (HGF), and mast/stem cell growth factor (M/SCGF) showed light-depended transcription, while those related to insulin-like growth factor mainly reached their highest expressions after short-term light exposure (Fig. 4c & d).

### Structural and functional change of *O. rhodostigmatus* MC1R

**Fig. 5:**
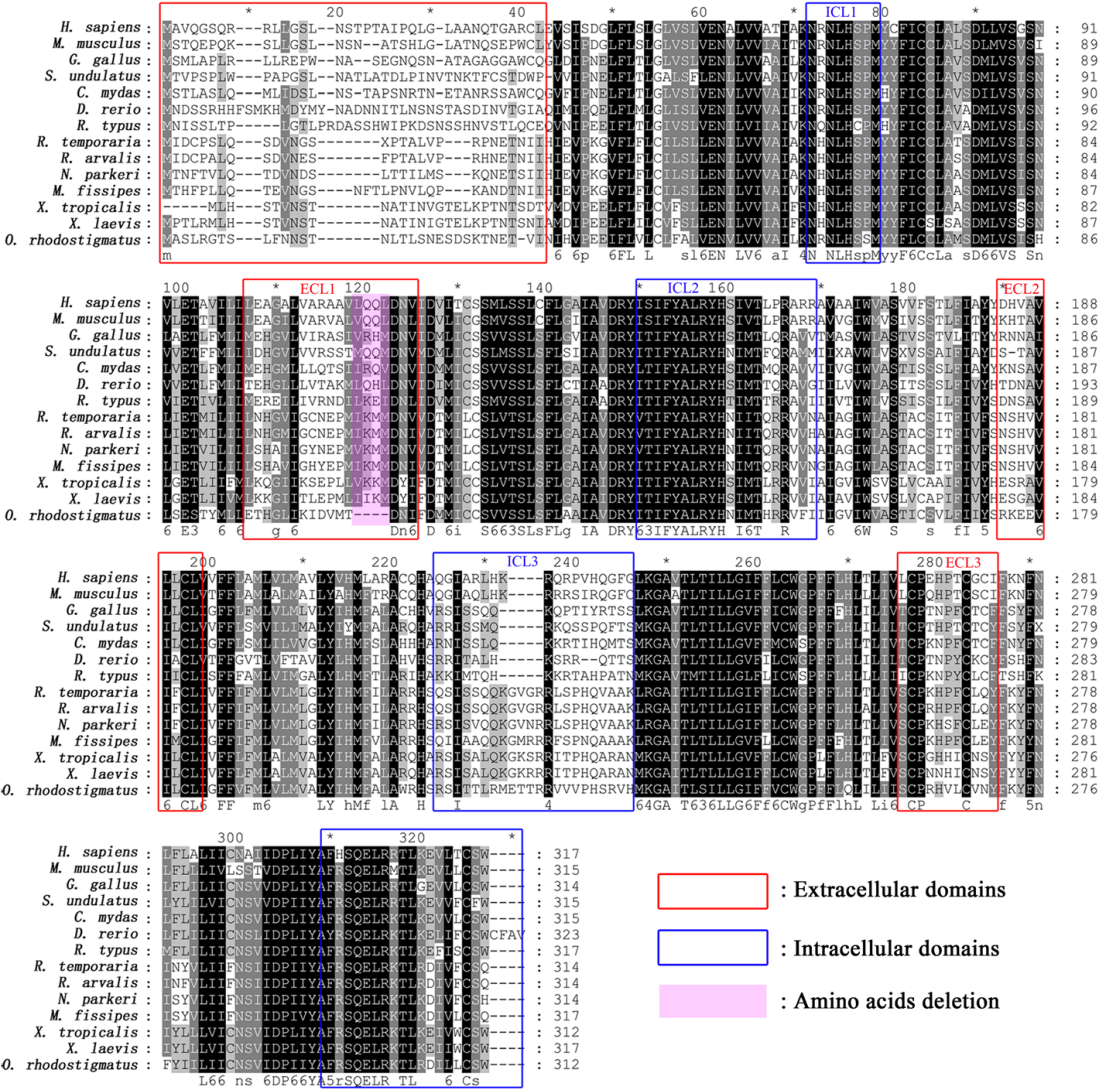
Sequence alignment of MC1R genes. Amino acids outside the frames are speculative transmembrane domains according to the deduced Human MC1R structure [24]

The sequences of *O. rhodostigmatus* α-MSH, MC1R, MC4R, MC5R, MCHR, agouti, TYR, TYRP1 and DCT were aligned with that of fishes, other amphibians, reptiles, birds and mammals. Prominent difference was identified only in *O. rhodostigmatus* MC1R. The length of ECL1 of MC1R was conserved across vertebrates, however, a deletion of four amino acids in this region was detected in its *O. rhodostigmatus* MC1R (Fig. 5). Such a deletion changed the structure of the M/EJTD and resulted in a longer third transmembrane helix in *O. rhodostigmatus* MC1R than that of other species, as suggested by the 3D models (Additional file 7: Figure S2).

**Fig. 6:**
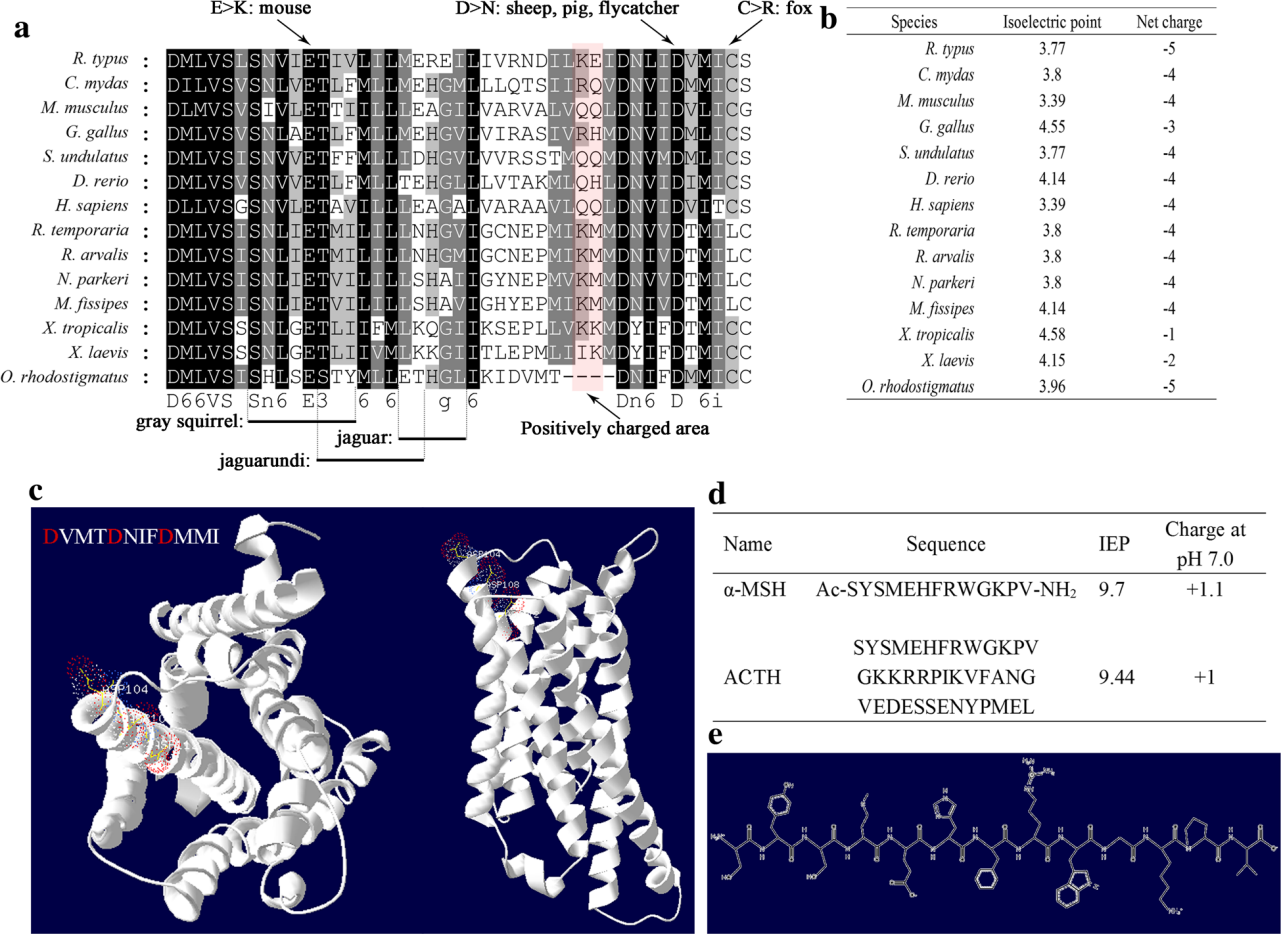
Sequence and structure change of MC1R and its implications in pigmentation of *O. rhodostigmatus*. **a**, Sequence analysis on the M/EJTD. Pink background indicates sites frequently occupied by basic residues. Missense mutations and amino acid deletions resulting melanic phenotypes were showed. **b**, Charges of this sequence fragment. **c**, 3D model of *O. rhodostigmatus* MC1R, with tandem aspartates highlighted. **d**, Sequences and charges of major agonists of MC1R. **e**, Structure of α-MSH

Since the charged amino acids responsible for agonist binding and constitutive activation of MC1R, we further analyzed the charge property of this region. Interestingly, fragment deletion in *O. rhodostigmatus* MC1R covered the sites with high frequency of positively charged amino acids (Fig. 6a), and the net charge of this region is − 5 in *O. rhodostigmatus*, more negatively charged than other frogs (Fig. 6b). On the other hand, fragment deletion in *O. rhodostigmatus* MC1R resulted in a “Asp× × ×Asp× × ×Asp× × ×” sequence mode, and these three negatively charged aspartate residues were aligned towards the ligand pocket of MC1R (Fig. 6c), quite different with the distribution of aspartate residues in other MC1Rs (Additional file 7: Figure S2). In combination with the elongated third helix of MC1R, these three stereo-tandem aspartate residues likely performed as a trap for positively charged ligands. Interestingly, both MSH and ACTH, who are positively charged at pH 7.0 (Fig. 6d-e) with isoelectric points (IEP) were higher than 9.0.

## Discussion

### Molecular processes involved in light-induced rapid skin darkening

Though the genes involved in synthesis of melanin, carotenoids and pteridines are all transcribed in *O. rhodostigmatus* tadpoles, only melanin-related genes showed increased transcription with light exposure (Fig. 2). It suggested that only melanocytes were involved in skin darkening in *O. rhodostigmatus* tadpoles, as melanin has a superiority on UV-light absorption in comparing with carotenoids and pteridines. Both PCC and MCC may be involved in melanin-based pigmentation. Generally speaking, PCC is more active and rapid than MCC in response to environmental brightness in fish and amphibians [1], and in most studied cases, activation of MCC is always accompanied by PCC, as factors inducing MCC can also activate PCC [53]. However, in *O. rhodostigmatus* tadpoles, genes involved in MCC, or more exactly melanogenesis and melanocyte proliferating, increased with skin darkening, while those involved in PCC did not (Fig. 2). Though transcriptional activation of related genes is not necessarily for PCC in existing melanocytes, increased expression of these genes along with melanocyte proliferating should be expected, if PCC contributes to skin darkening. These results suggested that MCC contributes to the rapid darkening of *O. rhodostigmatus* tadpoles. PCC in *O. rhodostigmatus* may be not as responsible to light as it in other amphibians, at least in aspect of pigment dispersion. It may be resulted from long-term adaptation to darkness, where PCC system is less required for fine-tuning skin color.

On the contrary, it is surprising that the light-induced MCC of *O. rhodostigmatus* tadpoles is so robust to darken their skin color within 15 h. Either expression or genetic mutations of genes involved in melanin synthesis (i.e. TYR, TYRP1 and DCT) may be responsible. However, no significant functional changes on amino acid sequences were identified in these genes of *O. rhodostigmatus*, in comparison with other vertebrates. Besides, constitutive enhancement on enzyme activities, facilitated by genetic mutations, go against the transparent phenotype of dark living *O. rhodostigmatus* tadpoles, whose TYR, TYRP1 and DCT genes are also transcribed, even at low levels (Fig. 2a). Therefore, light-induced gene expression should be a dominate contributor to this rapid darkening phenomenon in these tadpoles, as supported by the significant transcriptional upregulation of genes for melanin synthesis (Fig. 2a). Upstream regulating on transcription of these genes likely provides critical insight into the potential optical switch on pigmentation in these non-obligate cave dwellers.

### Regulation and genetic basis of rapid skin darkening

MITF is the critical transcriptional factor activating melanocyte differentiation and melanogenesis in vertebrates [32, 54]. The activity of MITF in melanogenesis is regulated at transcription and posttranslational modification. In *O. rhodostigmatus* tadpoles, the abundance of MITF transcripts was as low as undetectable by our RNA-seq programmes in lack of light, while rapid and significant transcriptional upregulation was induced by light exposure (Fig. 3). It is interesting that light-induced transcription of MITF peaked after short-term exposure, different from the variation pattern of enzymes in melanin synthesis. It suggested that the transient transcriptional impulse of MITF was intensive enough for initiating and maintaining melanogenesis in *O. rhodostigmatus* tadpoles.

Transcription of MITF is activated by cAMP signal mediated by α-MSH (or other analogous signals) and its receptor MC1R. The genetic change of MC1R should be responsible for the rapid and intensive transcription of MITF. It is known that the M/EJTD of MC1R is negatively charged and responsible for the binding of positively changed ligands, including α-MSH and ACTH (Fig. 6d-e). The conserved glutamate in the second transmembrane helix 89E in *O. rhodostigmatus* MC1R) and the second conserved aspartate in the third transmembrane helix (112D in *O. rhodostigmatus* MC1R) are responsible for ligands binding, as replacement of these two negatively charged amino acid residues with either positively charged ones or neutral ones would reduce the responsiveness of MC1R to α-MSH [40, 42]. Besides, replacement the aspartate residue at this position with lysine, asparagine, or valine decreases α-MSH binding affinity 10–100 fold [40]. Similarly, replacement of 89E with a basic amino acid residue would also decrease α-MSH binding affinity [40]. It suggested that negative charges in this region would reinforce the binding capacity of α-MSH. For *O. rhodostigmatus*, the stereo-tandem negative charges in the elongated third transmembrane helix likely performs as a ligands trap, which would enhance the ligands trapping and binding capacities of MC1R, as well as its responsiveness to ligands. It means that lower concentration of α-MSH is required to induce ligands-dependent activation of MC1R in *O. rhodostigmatus* than in other frogs. Besides, though it has not been reported that introducing extra acidic amino acid residues in membrane/extracellular junction of the third transmembrane domain would improve the maximum activity of MC1R, replacement of 112D with lysine, asparagine, or valine could reduce the maximum activity of MC1R. Similar outcomes were resulted from replacement of 116C and 89E with the basic asparagine [40, 42]. It seems that the maximum activity of MC1R is also related to the charge characteristic of this region, and negative charges likely improve the maximum activity. Taken together, it can be speculated that more efficiency and intensive activation of MC1R would be induced by α-MSH in *O. rhodostigmatus* than in other frogs, which should be a prime genetic basis for rapid MCC.

The posttranslational modification might also play a role in rapid skin darkening by amplifying the MITF signal. Phosphorylation is required for MITF activation in MAPK pathway depended manner [24–26], and activation of MAPK pathway is a common and immediate downstream event of GFR activation in proliferating melanocyte [55]. In *O. rhodostigmatus* tadpoles, light exposure induced significant transcriptional upregulation of numerous growth factor related genes and core components involved in MAPK pathway (Fig. 4). It was possible that light-induced transcriptional activation of growth signal and MAPK pathway was beneficial to MITF phosphorylation in *O. rhodostigmatus* tadpoles, and thus facilitating rapid skin darkening.

In addition, growth signals and their downstream signal pathways, typically MAPK and PI3K-Akt signal pathways, facilitate melanocyte proliferation in MITF-independent manner [22]. Synergistic effect of growth factors has been widely reported in melanocyte proliferation. For human melanocytes, FGFs, HGFs, M/SCGFs are strong synergistic mitogens, α-MSH and ACTH are weak mitogens, while EGFs and VEGFs are ineffective mitogens, and at least two stimulators are required for their proliferating [54]. In *O. rhodostigmatus* melanocyte, EGFs, PDGFs, FGFs, HGFs and M/SCGFs related genes were synchronously upregulated, covering all the three strong synergistic mitogens identified for human melanocytes. Therefore, a strong proliferative stimulus can be expected from their synergistic effects. The strong synergistic effect of growth signal may be one of the reasons for the rapid skin darkening, if it is proved to be associated with melanocyte proliferation in *O. rhodostigmatus*.

### Genetic basis of pigment regression

In contrast to rapid MCC, cave adaptation requires reduced MC1R activity to keep their transparent skin color. *Astyanax mexicanus* is the most studied cavefish, whose cave population showed obvious pigment regression in comparison with surface populations. In cave population collected from Pachon, MC1R variant with 2 bp deletion in coding region resulted in non-functional product has been identified [9]. While in cave population from Yerbaniz, MC1R variant with a point mutation in its coding region has been identified, and it resulted replacement of R164 (R151 in *O. rhodostigmatus*) with cysteine residue [9], which also found in humans with red hair and pale skin [52]. Both mutations are associated with pigment regression in cave adaptive populations. They are located out of the ligands binding region and reduce the activity of MC1R whether constitutively or ligands dependently. Besides MC1R, defects in genes involved in melanin biosynthesis (i.e., OCA2 and TYR) were also identified in cave dwellers [8, 11, 12, 34]. Obviously, these types of mutations strengthen the cave adaptability at the expense of their adaptability to bright environment. Unlike cavefish, *O. rhodostigmatus* should possess strong capacity of light-induced MC1R activation for lifehistory after metamorphosis, and so the mechanisms underlying their cave adaptation should be different from that of cavefish.

Though replacement of acidic amino acid residues with neutral or basic ones, or replacements of neutral residues with basic ones, in membrane/extracellular junction of the second and third transmembrane domains would reduce the affinity and responsiveness of MC1R to α-MSH, it could also enhance the constitutive activation of MC1R and thus results in dark phenotypes [40]. For example, the replacement of 89E (in *O. rhodostigmatus*) with asparagine was identified in the melanic mouse [42], chicken and bananaquits [43, 44], the replacement of 112D with glutamine was identified in the melanic sheep [45], pig [46] and flycatcher [41], and the replacement of 116C with asparagine was identified in the dark color fox [46]. Additionally, amino acids deletion in this region was also widely reported in MC1R of animals with melanic phenotypes (Fig. 6a) [48–50], and these deletions always cover the conserved or high-frequency sites for acidic residues. It has been proven that positive charged residues in this region are necessary for constitutive activation of MC1R in manner of ligands mimicking [40, 42]. Accordingly, it can be speculated that introducing extra acidic residues, or replacements of basic residues with neutral and acidic ones, which is exactly the situation in *O. rhodostigmatus*, would weaken the constitutive activity of MC1R. It provided a genetic explanation for the transparent phenotype of *O. rhodostigmatus* tadpoles. It also explained why permanent pigment regression or pale phenotypes granted by acidifying the M/EJTD of MC1R has not been identified in animals [9, 51, 52], even melanistic phenotypes granted by basic residues introducing and/or acid residues eliminating in these regions are common in vertebrates [40–50].

**Fig. 7:**
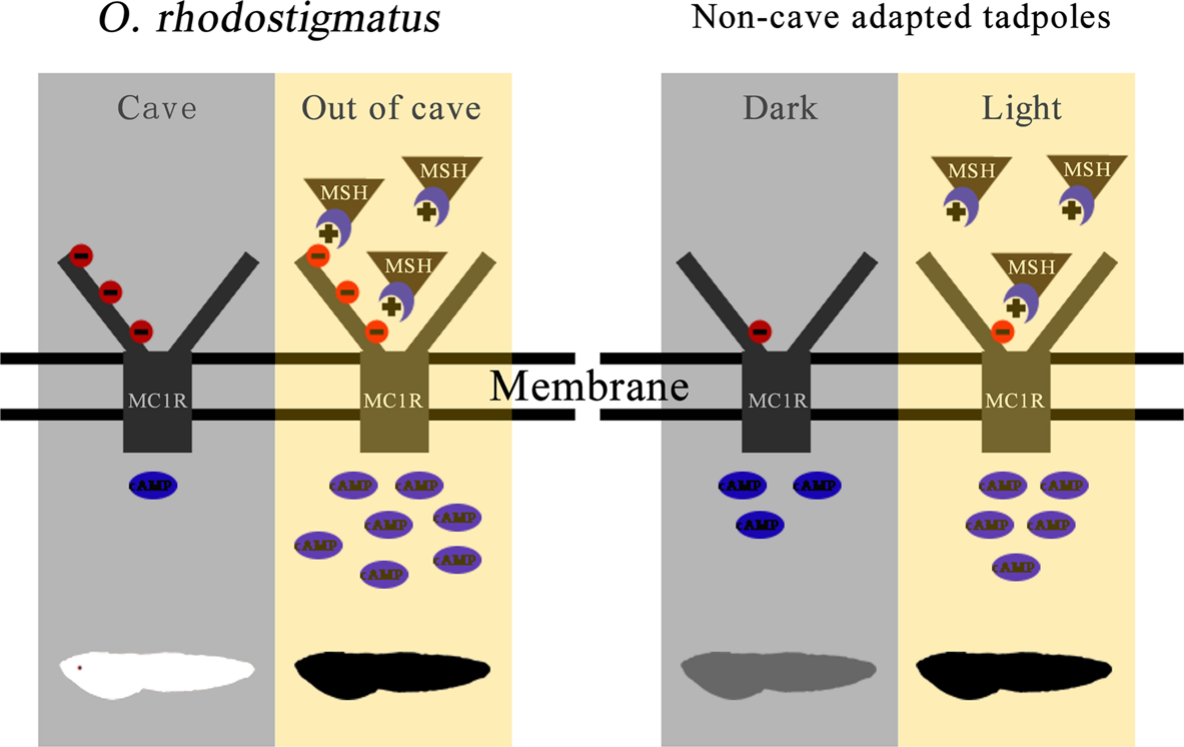
Deduced mechanism by which *O. rhodostigmatus* MC1R coordinates pigment regression in darkness and rapid melanogenesis (or other types of pigment production) in response to light exposure

Overall, the fragment deletion and extra acidic amino acid residues in the ligands binding domain of MC1R likely reduced the constitutive activity of MC1R, but reinforced its ligands-dependent activity, which might contribute to pigment regression for cave adaptation and rapid MCC for transforming of life history, respectively (Fig. 7).

## Conclusion

Tadpoles of *O. rhodostigmatus* are non-obligate cave dwellers, who keep transparent phenotype in caves but rapidly darken in light within 15 h. Using comparative transcriptomics, we found that the melanocyte MCC (including melanogenesis and melanocytes proliferation) was responsible for the rapid skin darkening in *O. rhodostigmatus* tadpoles. As the most prominent change, light-induced transcriptional activation of growth signals (including growth factor signals, MAPK signal pathways and PI3K-Akt signal pathways) may facilitate the rapid MCC in *O. rhodostigmatus* tadpoles. The most amazing found here is that an in-frame deletion of four amino acids in the M/EJTD of *O. rhodostigmatus* MC1R, the receptor for melanogenesis signal, was identified. This mutation increases the negative charge of the ligand pocket of MC1R and results in the stereo-tandem of three aspartate residues aligning towards its ligand pocket. The ligand pocket of *O. rhodostigmatus* MC1R resembles a trap for positively charged ligands (α-MSH and ACTH) and likely increases the ligands-dependent activity of MC1R, providing an explanation for the rapid MCC of *O. rhodostigmatus* in light. Meanwhile, increased negative charge of ligand pocket likely decreased the constitutive activity of MC1R, supporting the transparent phenotype of the cave-dwelling tadpoles. Therefore, genetic mutations of MC1R explains, at least to some extent, how the pigmentation system of *O. rhodostigmatus* coordinates the capacity of rapid melanogenesis (or other types of pigment production) and pigment regression, a couple of seemingly contradictory coloration requirements. To our knowledge, this is the first study that reported the association between pigmentation phenotype adaptation and MC1R mutations in amphibians and/or in the non-obligate cave dwellers.

## Methods

### Sample collection and treatment

Tadpoles of *Oreolalax rhodostigmatus* were collected in a karst cave of Shizhu County, Chongqing City, China in April 2017. In the cave, a stream runs from inside to outside, with stable water temperature of 15.3 °C. When Beijing time is five a.m., a total of 13 “transparent” tadpoles were collected simultaneously from a pool in the dark zone of the cave, and all of them were identified at their Gosner stage of 25 [56]. Once collected, four of them were immediately anaesthetized and sacrificed to collect eyeballs, liver (0.5 cm^3^), dorsal skin (1 cm^2^) and half tail (3 cm length). Tissues of each tadpole were all together placed into one tissue tube as one sample, and then preserved in liquid nitrogen. These four samples were defined in a “Control group”. The rest tadpoles were raised in the cage immersed in the pool at the entrance of the cave, where is light-accessible, and after 8 p.m. a broad-spectrum fluorescent lamp was used to imitate sunlight at night. Four hours later, six tadpoles were anaesthetized and sacrificed to collect tissues as described above which were defined as a “Short-term exposed group”, and tissues of each tadpole were all together placed into one tissue tube as one sample. The rest three tadpoles were similarly collected after 30 h of light exposure, and these samples were defined as a “Long-term exposed group”.

### cDNA library construction and Illumina sequencing

Total RNA of each sample was extracted and purified using Trizol (Invitrogen, Carlsbad, CA, USA) following the manufacturer’s instructions. After purified with poly-T oligo-attached magnetic beads, the mRNAs were fragmented. First-strand cDNA was synthesized using random hexamer primers and M-MuLV Reverse Transcriptase (RNase H−). Second-strand cDNA synthesis was subsequently performed using DNA Polymerase I and RNase H. The remaining overhangs were converted into blunt ends via exonuclease/polymerase activities. After adenylation of the 3′ ends of the DNA fragments, NEBNext adaptors with a hairpin loop structure were ligated to prepare for hybridization. To preferentially select cDNA fragments of 150–200 bp in length, the library fragments were purified with AMPure XP system (Beckman Coulter, Beverly, USA). Then, 3 μl USER Enzyme (NEB, USA) was used with size-selected, adaptor-ligated cDNA at 37 °C for 15 min followed by 5 min at 95 °C before PCR. Then, PCR was performed with a Phusion High-Fidelity DNA polymerase, universal PCR primers and Index (X) primer. Finally, PCR products were purified (AMPure XP system), and library quality was assessed on the Agilent Bioanalyzer 2100 system. The clustering of the index-coded samples was performed on a cBot Cluster Generation System using the TruSeq PE Cluster Kit v3-cBot-HS (Illumina) according to the manufacturer’s instructions. After cluster generation, the library preparations were sequenced on an Illumina HiSeq 4000 platform by NovoGene (Beijing), and paired-end reads were generated.

### De novo transcriptome assembly and transcriptome annotation

The reads quality was verified using FastQC (version 0.10.0) software. Reads containing adapters, reads containing poly-N and low-quality reads were removed. The total clean reads from all libraries were assembled de novo using Trinity as a reference transcriptome. The resulted unigenes were annotated by querying databases NR, NT and Swiss-Prot with an E-value threshold of 1.0E-5 and KOG with an E-value threshold of 1.0E-3. Then, Blast2GO software was used to obtain GO annotations defined by molecular function, cellular component and biological process ontologies. Pathway assignments were determined based on the KEGG database using BLASTX with an E-value threshold of 1.0E-5.

### Validity assessment of transcriptome data

The expression level of each unigene was expressed as fragments per kilobase of exon model per million mapped reads (FPKM). Pearson correlations of gene expression levels between samples were calculated to assess the validity of transcriptome data. Generally speaking, valid data should have higher intra-group correlations than inter-group ones.

### Analysis of differentially expressed genes

The analysis flow was showed in Fig. 1 H. Briefly, unigenes, whose expression levels showed upregulation with light exposure (false discovery rate < 0.05, one-way ANOVA & BHfdr), were considered to be light inducible genes. On this basis, those showed significant upregulations in pairwise comparisons, “short-term exposed vs control” and “long-term exposed vs short-term exposed”, were stricter light inducible genes. These unigenes were uploaded to Kobas 3.0 (http://kobas.cbi.pku.edu.cn/index.php) for enrichment analysis [57]. Information of unigenes used in analysis and figures was summarized in Additional file 8: Table S6.

### Sequence comparison and phylogenetic analyses

Sequences of targeted genes were retrieved from Genbank or our transcriptome database. N-J tree was built on MEGA7 with default parameters. Sequence alignment was performed on Clustal X2, and further edit was performed on GeneDoc.

### Prediction of protein 3D models

3D models of MC1R proteins were predicted on Swiss-model server (https://www.swissmodel.expasy.org/) with “Chimera protein of human 5-hydroxytryptamine receptor 1B (ID: 4iaq.1)” as model [58]. Analysis of 3D models was performed on Swiss PDB Viewer.

### Analysis of peptide property

Peptide physicochemical property was analyzed on PepDraw online server (http://www.tulane.edu/~biochem/WW/PepDraw/index.html).

## Additional files

Additional file 1: **Table S1.** Summary of sequencing quality. (XLSX 9 kb)

Additional file 2: **Figure S1.** Length distribution of transcripts and unigenes. (PDF 4 kb)

Additional file 3: **Table S2.** Unigene annotation details. (XLSX 49576 kb)

Additional file 4: **Table S3.** Summary of FPKM. (XLSX 20703 kb)

Additional file 5: **Table S4.** Summary of enrichment based on light-inducible genes (1662). (XLSX 46 kb)

Additional file 6: **Table S5.** Summary of enrichment based on stricter light-inducible genes (213). (XLSX 32 kb)

Additional file 7: **Figure S2.** 3D-model of MC1R in frogs. Models are built based on human 5-hydroxytryptamine receptor. Red arrows indicate ending point of TMH3 in extracellular side. (PDF 11742 kb)

Additional file 8: **Table S6.** Information of unigenes used in analysis and figures. (XLSX 538 kb)

## Abbreviations

ACTH: Adrenocorticotropic hormone
ASIP: Agouti signal peptide
BCO1: Beta, beta-carotene 15,15′-monooxygenase
BCO2: Beta, beta-carotene 9′,10′-oxygenase
DCT: Dopachrome tautomerase
DHPR: Dihydropteridine reductase
EGF: Epidermal growth factor
FGF: Fibroblast growth factor
GCH1: GTP cyclohydrolase 1
GCHFR: GTP cyclohydrolase 1 feedback regulatory protein
GFR: Growth factor receptor
HGF: Hepatocyte growth factor
M/SCGF: Mast/stem cell growth factor
MC1R: Melanocortin-1 receptor
MCC: Morphological color change
MCH: Melanin-concentrating hormone
MCHR: MCH receptor
MELANA: Melanoma antigen recognized by T-cells 1 isoform X1
MITF: Microphthalmia-associated transcription factor
PCC: Physiological color change
PDGF: Platelet-derived growth factor
PMEL: Premelanosome protein precursor
POMC: Precursor proopiomelanocortin protein
SPR: Sepiapterin reductase
TYR: Tyrosinase
TYRP1: Tyrosinase-related protein 1
XDH/XOD: Xanthine dehydrogenase/oxidase
α-MSH: alpha-Melanocyte-stimulating hormone

## References

[1] Nilsson Skold H, Aspengren S, Wallin M (2013). Rapid color change in fish and amphibians - function, regulation, and emerging applications. Pigment cell & melanoma research.

[2] Mills MG, Patterson LB (2009). Not just black and white: pigment pattern development and evolution in vertebrates. Semin Cell Dev Biol.

[3] Hansen RM, Fulton AB, Harris SJ (1986). Background adaptation in human infants. Vis Res.

[4] Logan DW, Burn SF, Jackson IJ (2006). Regulation of pigmentation in zebrafish melanophores. Pigment Cell Res.

[5] Fulton AB (1983). Background adaptation in RCS rats. Invest Ophthalmol Vis Sci.

[6] Leclercq E, Taylor JF, Migaud H (2009). Morphological skin colour changes in teleosts. Fish Fish.

[7] Jeffery WR, Li M, Parkhurst A, Bilandžija H (2014). Pigment regression and albinism in *Astyanax* cavefish.

[8] Yang J, Chen X, Bai J, Fang D, Qiu Y, Jiang W, Yuan H, Bian C, Lu J, He S (2016). The *Sinocyclocheilus* cavefish genome provides insights into cave adaptation. BMC Biol.

[9] Gross JB, Borowsky R, Tabin CJ (2009). A novel role for Mc1r in the parallel evolution of depigmentation in independent populations of the cavefish *Astyanax mexicanus*. PLoS Genet.

[10] Protas M, Jeffery WR (2012). Evolution and development in cave animals: from fish to crustaceans. Wiley Interdiscip Rev Dev Biol.

[11] McCauley DW, Hixon E, Jeffery WR (2004). Evolution of pigment cell regression in the cavefish *Astyanax*: a late step in melanogenesis. Evolution & Development.

[12] Protas ME, Hersey C, Kochanek D, Zhou Y, Wilkens H, Jeffery WR, Zon LI, Borowsky R, Tabin CJ (2006). Genetic analysis of cavefish reveals molecular convergence in the evolution of albinism. Nat Genet.

[13] Bilandžija H, Ma L, Parkhurst A, Jeffery WR (2013). A potential benefit of albinism in *Astyanax* cavefish: downregulation of the oca2 gene increases tyrosine and catecholamine levels as an alternative to melanin synthesis. PLoS One.

[14] Fei L, Ye C, Jiang J (2012). Colored atlas of Chinese amphibians and their distributions.

[15] Liu J (2010). Ontogenesis and primary ecological study of *Oreolalax rhodostigmatus*. Bulletin of Biology.

[16] Shen Y, Gu Q, Gu Z, Mao H (2014). *Oreolalax rhodostigmatus* in the North-Western Hunan province: the cave life and the characteristics of the growth and development of its tadpoles. Life Science Research.

[17] Ligon RA, McCartney KL (2016). Biochemical regulation of pigment motility in vertebrate chromatophores: a review of physiological color change mechanisms. Current Zoology.

[18] Kelman EJ, Tiptus P, Osorio D (2006). Juvenile plaice (*Pleuronectes platessa*) produce camouflage by flexibly combining two separate patterns. J Exp Biol.

[19] Rhodes SB, Schlupp I (2012). Rapid and socially induced change of a badge of status. J Fish Biol.

[20] Bertolesi GE, Song YN, Atkinson-Leadbeater K, Yang JJ, McFarlane S (2017). Interaction and developmental activation of two neuroendocrine systems that regulate light-mediated skin pigmentation. Pigment cell & melanoma research.

[21] Henning F, Jones JC, Franchini P, Meyer A (2013). Transcriptomics of morphological color change in polychromatic *Midas cichlids*. BMC Genomics.

[22] Levy C, Khaled M, Fisher DE (2006). MITF: master regulator of melanocyte development and melanoma oncogene. Trends Mol Med.

[23] Levy C, Khaled M, Robinson KC, Veguilla RA, Chen PH, Yokoyama S, Makino E, Lu J, Larue L, Beermann F (2010). Lineage specific transcriptional regulation of DICER by MITF in melanocytes. Cell.

[24] Garcia-Borron JC, Abdel-Malek Z, Jimenez-Cervantes C (2014). MC1R, the cAMP pathway, and the response to solar UV: extending the horizon beyond pigmentation. Pigment cell & melanoma research.

[25] Gilchrest BA, Park H-Y, Eller MS, Yaar M (1996). Mechanisms of ultraviolet light-induced pigmentation. Photochem Photobiol.

[26] Sugimoto M (2002). Morphological color changes in fish: regulation of pigment cell density and morphology. Microsc Res Tech.

[27] Mellgren EM, Johnson SL (2004). A requirement for kit in embryonic zebrafish melanocyte differentiation is revealed by melanoblast delay. Dev Genes Evol.

[28] Kumasaka M, Sato S, Yajima I, Goding CR, Yamamoto H (2005). Regulation of melanoblast and retinal pigment epithelium development by *Xenopus laevis* Mitf. Dev Dyn.

[29] Kumasaka M, Sato H, Sato S, Yajima I, Yamamoto H (2004). Isolation and developmental expression of Mitf in *Xenopus laevis*. Dev Dyn.

[30] Kawasaki A, Kumasaka M, Satoh A, Suzuki M, Tamura K, Goto T, Asashima M, Yamamoto H (2008). Mitf contributes to melanosome distributionand melanophore dendricity. Pigment cell & melanoma research.

[31] Fukuzawa T, Bagnara JT (1989). Control of melanoblast differentiation in amphibia by α-melanocyte stimulating hormone, a serum melanization factor, and a melanization inhibiting factor. Pigment Cell Res.

[32] Xia M, Chen K, Yao X, Xu Y, Yao J, Yan J, Shao Z, Wang G (2017). Mediator MED23 links pigmentation and DNA repair through the transcription factor MITF. Cell Rep.

[33] Santos ME, Baldo L, Gu L, Boileau N, Musilova Z, Salzburger W. Comparative transcriptomics of anal fin pigmentation patterns in cichlid fishes. BMC Genomics. 2016;17(1):712. 10.1186/s12864-016-3046-y PMC5012078 27600936

[34] Bilandžija H, Ćetković H, Jeffery WR (2012). Evolution of albinism in cave planthoppers by a convergent defect in the first step of melanin biosynthesis. Evolution & Development.

[35] Hubbard JK, Uy JA, Hauber ME, Hoekstra HE, Safran RJ (2010). Vertebrate pigmentation: from underlying genes to adaptive function. Trends in genetics : TIG.

[36] Nadeau NJ, Minvielle F, Mundy NI (2006). Association of a Glu92Lys substitution in MC1R with extended brown in Japanese quail (*Coturnix japonica*). Anim Genet.

[37] Takeuchi S, Suzuki H, Yabuuchi M, Takahashi S (1996). A possible involvement of melanocortin 1-receptor in regulating feather color pigmentation in the chicken. Biochim Biophys Acta.

[38] Ling MK, Lagerström MC, Fredriksson R, Okimoto R, Mundy NI, Takeuchi S, Schiöth HB (2003). Association of feather colour with constitutively active melanocortin 1 receptors in chicken. Eur J Biochem.

[39] Doucet SM, Shawkey MD, Rathburn MK, Jr MH, Montgomerie R (2004). Concordant evolution of plumage colour, feather microstructure and a melanocortin receptor gene between mainland and island populations of a fairy–wren. Proceedings Biological Sciences.

[40] Lu D, Vage DI, Cone RD (1998). A ligand-mimetic model for constitutive activation of the melanocortin-1 receptor. Mol Endocrinol.

[41] Uy JA, Moyle RG, Filardi CE, Cheviron ZA (2009). Difference in plumage color used in species recognition between incipient species is linked to a single amino acid substitution in the melanocortin-1 receptor. Am Nat.

[42] Benned-Jensen T, Mokrosinski J, Rosenkilde MM (2011). The E92K melanocortin 1 receptor mutant induces cAMP production and arrestin recruitment but not ERK cctivity indicating biased constitutive signaling. PLoS One.

[43] Robbins LS, Nadeau JH, Johnson KR, Kelly MA, Roselli-Rehfuss L, Baack E, Mountjoy KG, Cone RD (1993). Pigmentation phenotypes of variant extension locus alleles result from point mutations that alter MSH receptor function. Cell.

[44] Theron E, Hawkins K, Bermingham E, Ricklefs RE, Mundy NI (2001). The molecular basis of an avian plumage polymorphism in the wild: a melanocortin-1-receptor point mutation is perfectly associated with the melanic plumage morph of the bananaquit, *Coereba flaveola*. Current biology : CB.

[45] Våge DI, Klungland H, Lu D, Cone RD (1999). Molecular and pharmacological characterization of dominant black coat color in sheep. Mamm Genome.

[46] Kijas JMH, Wales R, Tornsten A, Chardon P, Moller M, Andersson L (1998). Melanocortin receptor 1 (MC1R) mutations and coat color in pigs. Genetics.

[47] Våge DI, Lu D, Klungland H, Lien S, Adalsteinsson S, Cone RD (1997). A non-epistatic interaction of agouti and extension in the fox, Vulpes vulpes. Nat Genet.

[48] Eizirik E, Yuhki N, Johnson WE, Menotti-Raymond M, Hannah SS, O'Brien SJ. Molecular Genetics and Evolution of Melanism in the Cat Family. Current biology. 2003;13(5):448-53. 10.1016/s0960-9822(03)00128-3 12620197

[49] Fontanesi L, Tazzoli M, Beretti F, Russo V (2006). Mutations in the melanocortin 1 receptor (MC1R) gene are associated with coat colours in the domestic rabbit (*Oryctolagus cuniculus*). Anim Genet.

[50] McRobie H, Thomas A, Kelly J (2009). The genetic basis of melanism in the gray squirrel (*Sciurus carolinensis*). The Journal of heredity.

[51] Dreger DL, Schmutz SM (2010). A new mutation in MC1R explains a coat color phenotype in 2 "old" breeds: saluki and afghan hound. The Journal of heredity.

[52] Raimondi S, Sera F, Gandini S, Iodice S, Caini S, Maisonneuve P, Fargnoli MC (2008). MC1R variants, melanoma and red hair color phenotype: a meta-analysis. Int J Cancer.

[53] Bertolesi GE, Hehr CL, Munn H, McFarlane S (2016). Two light-activated neuroendocrine circuits arising in the eye trigger physiological and morphological pigmentation. Pigment cell & melanoma research.

[54] Halaban R (2000). The regulation of normal melanocyte proliferation. Pigment Cell Res.

[55] El-Abaseri TB, Fuhrman J, Trempus C, Shendrik I, Tennant RW, Hansen LA (2005). Chemoprevention of UV light-induced skin tumorigenesis by inhibition of the epidermal growth factor receptor. Cancer Res.

[56] Gosner KL. A simplified table for staging anuran embryos and larvae with notes on identification. Herpetologica. 1960;16(3):183-90.

[57] Xie C, Mao X, Huang J, Ding Y, Wu J, Dong S, Kong L, Gao G, Li CY, Wei L. KOBAS 2.0: a web server for annotation and identification of enriched pathways and diseases. Nucleic Acids Res. 2011;39(Web Server issue):W316-322. 10.1093/nar/gkr483 PMC3125809 21715386

[58] Biasini M, Bienert S, Waterhouse A, Arnold K, Studer G, Schmidt T, Kiefer F, Cassarino TG, Bertoni M, Bordoli L. SWISS-MODEL: modelling protein tertiary and quaternary structure using evolutionary information. Nucleic Acids Res. 2014;2(Web Server issue):W252. 10.1093/nar/gku340 PMC4086089 24782522

