# Supplementary figures and images for: Transcriptomics reveals the molecular processes of light-induced rapid darkening of the non-obligate cave dweller Oreolalax rhodostigmatus (Megophryidae, Anura) and their genetic basis of pigmentation strategy
Source: BMC Genomics. 2018 May 31;19:422. doi: 10.1186/s12864-018-4790-y (PMC5984452; doi:10.1186/s12864-018-4790-y)

# Length Distribution

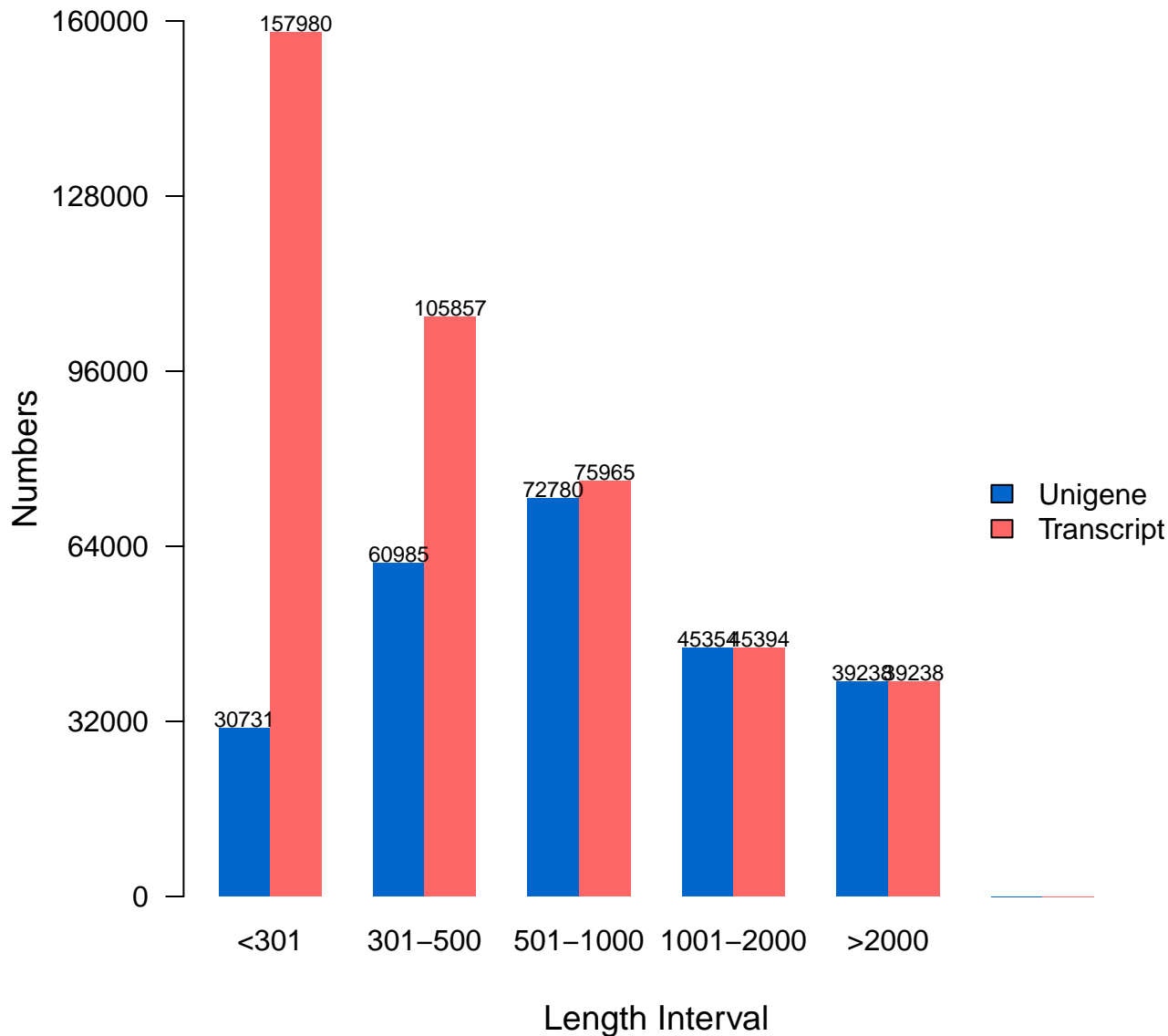

Supplement: Supplementary file 2 — Figure S1. Length distribution of transcripts and unigenes. (PDF 4 kb) [file 12864_2018_4790_MOESM2_ESM.pdf]

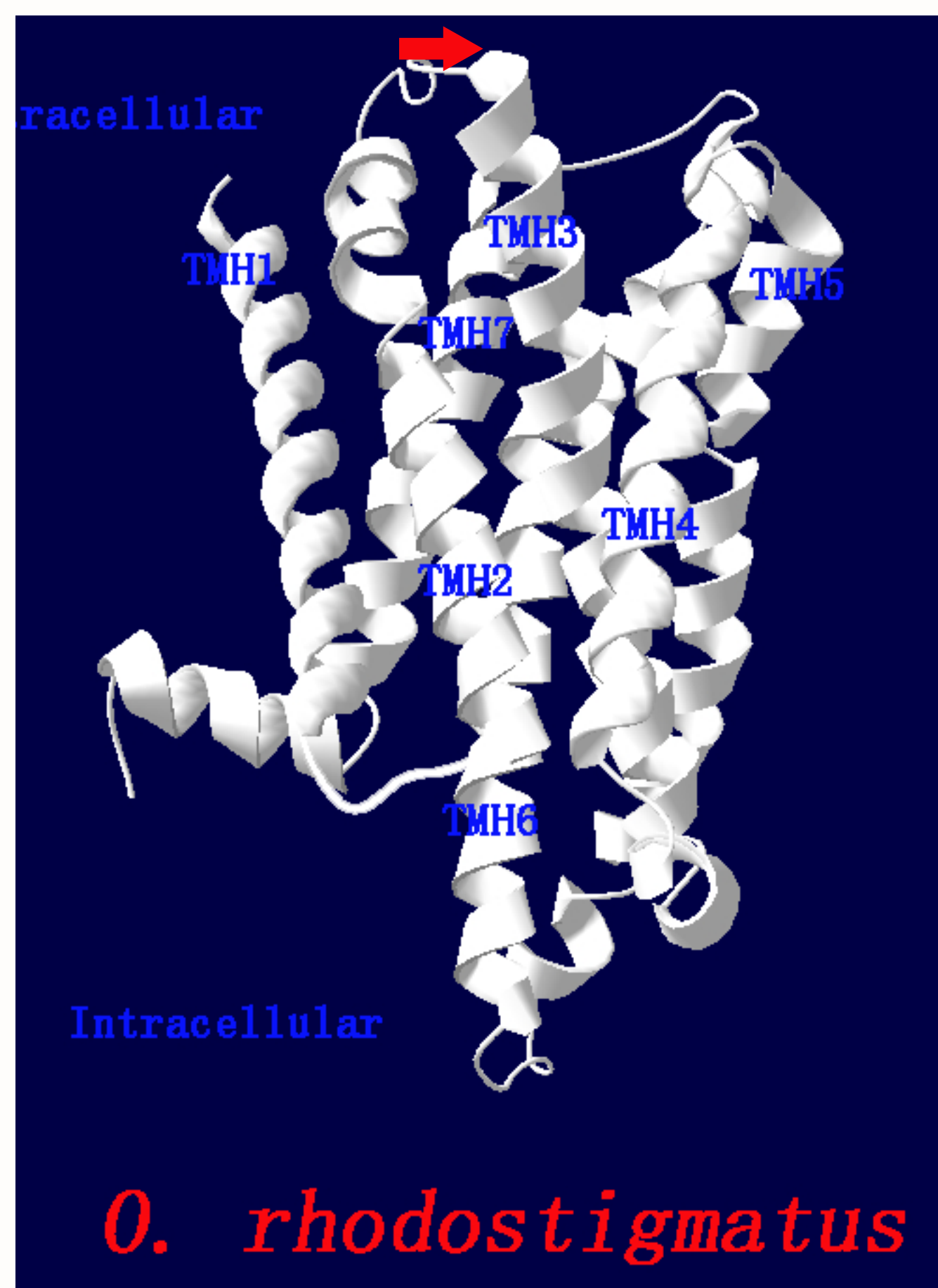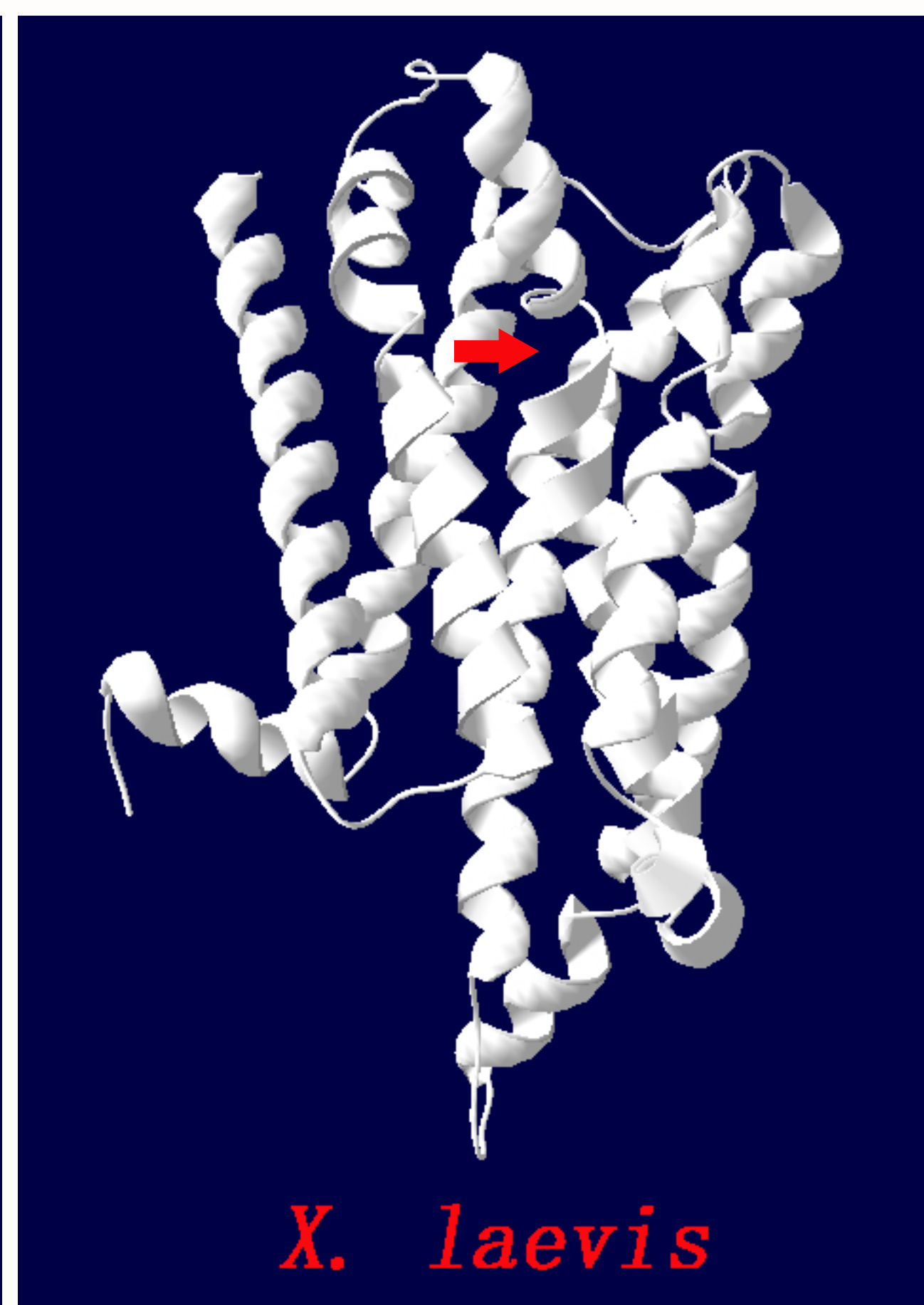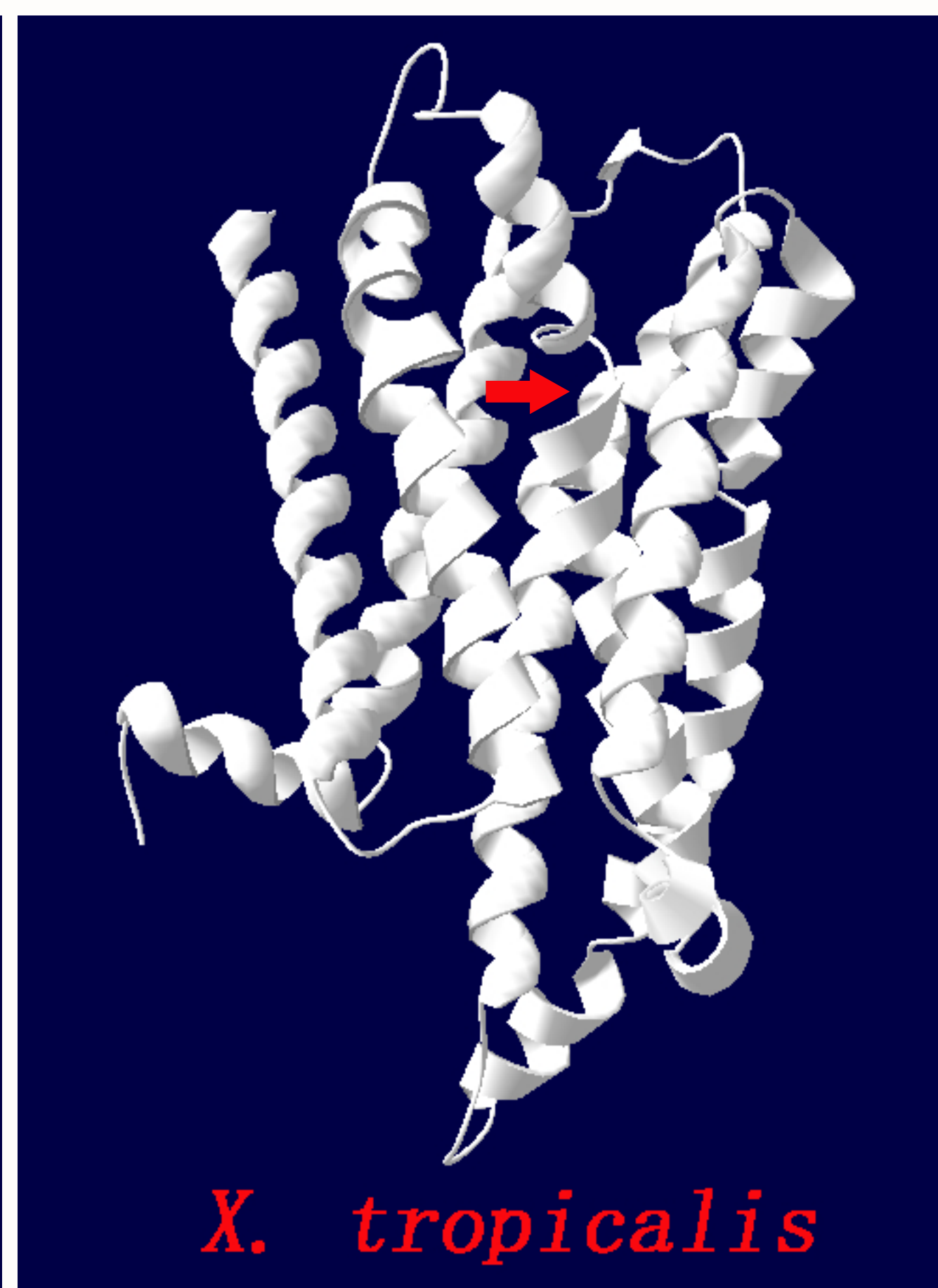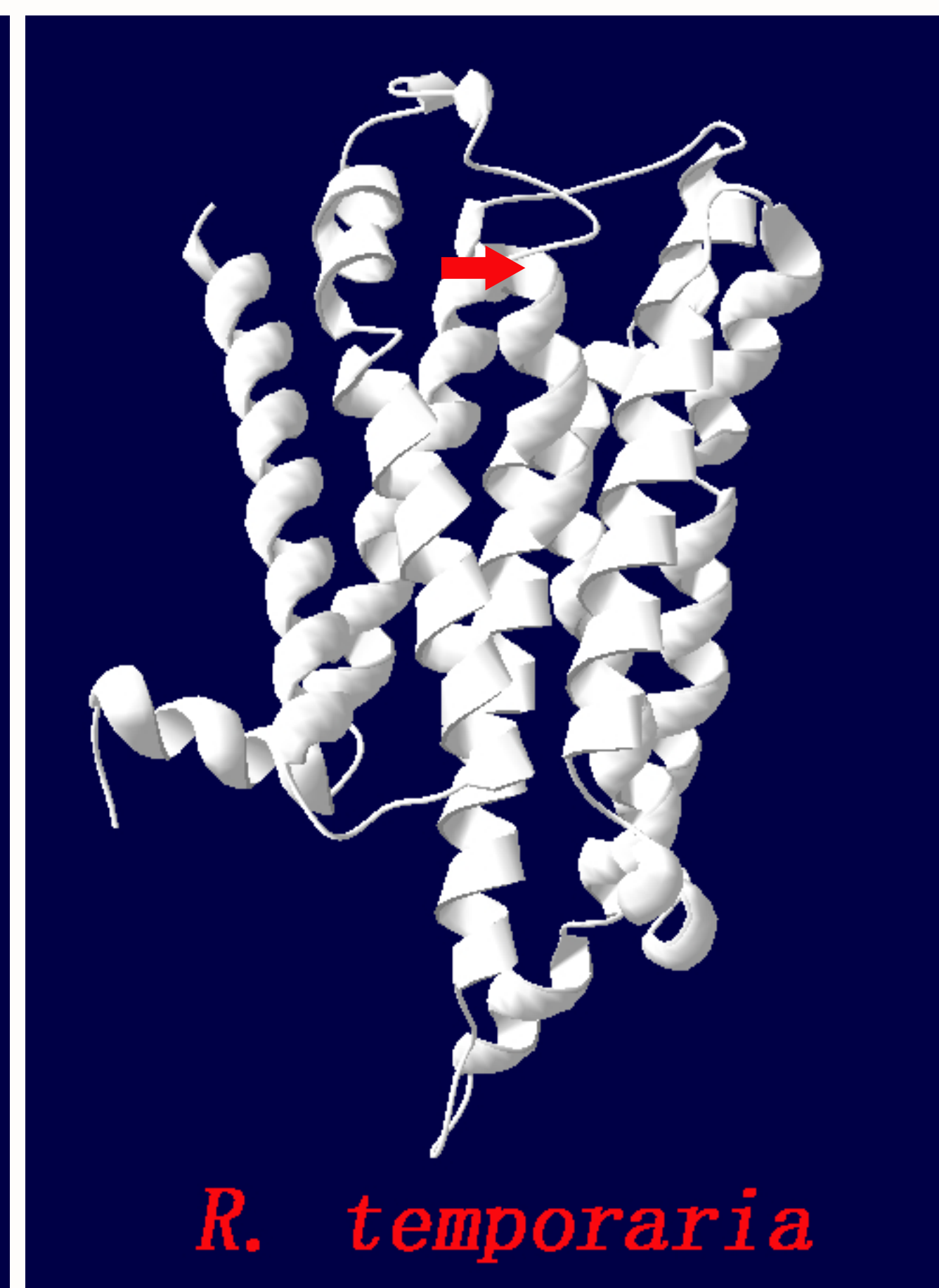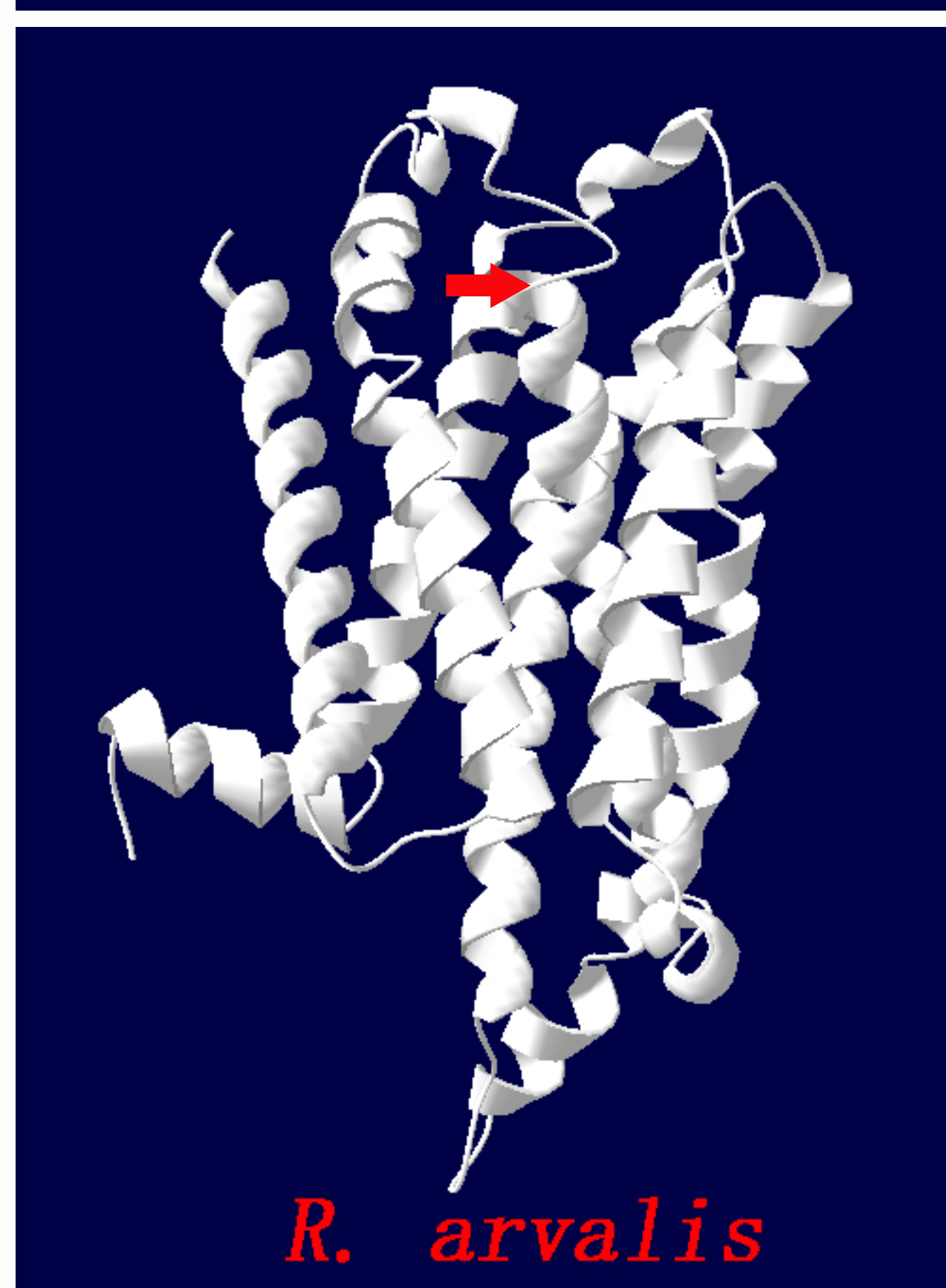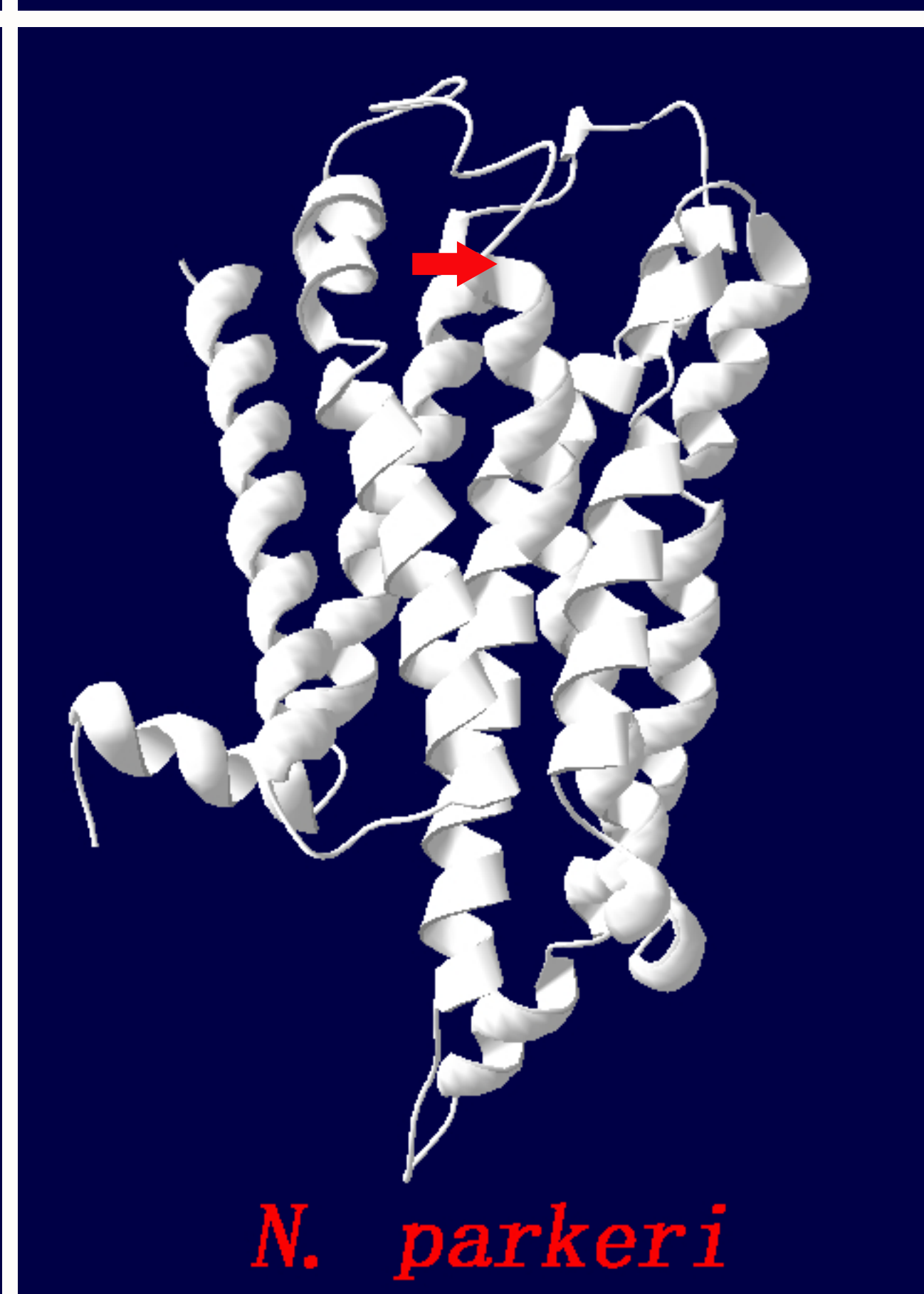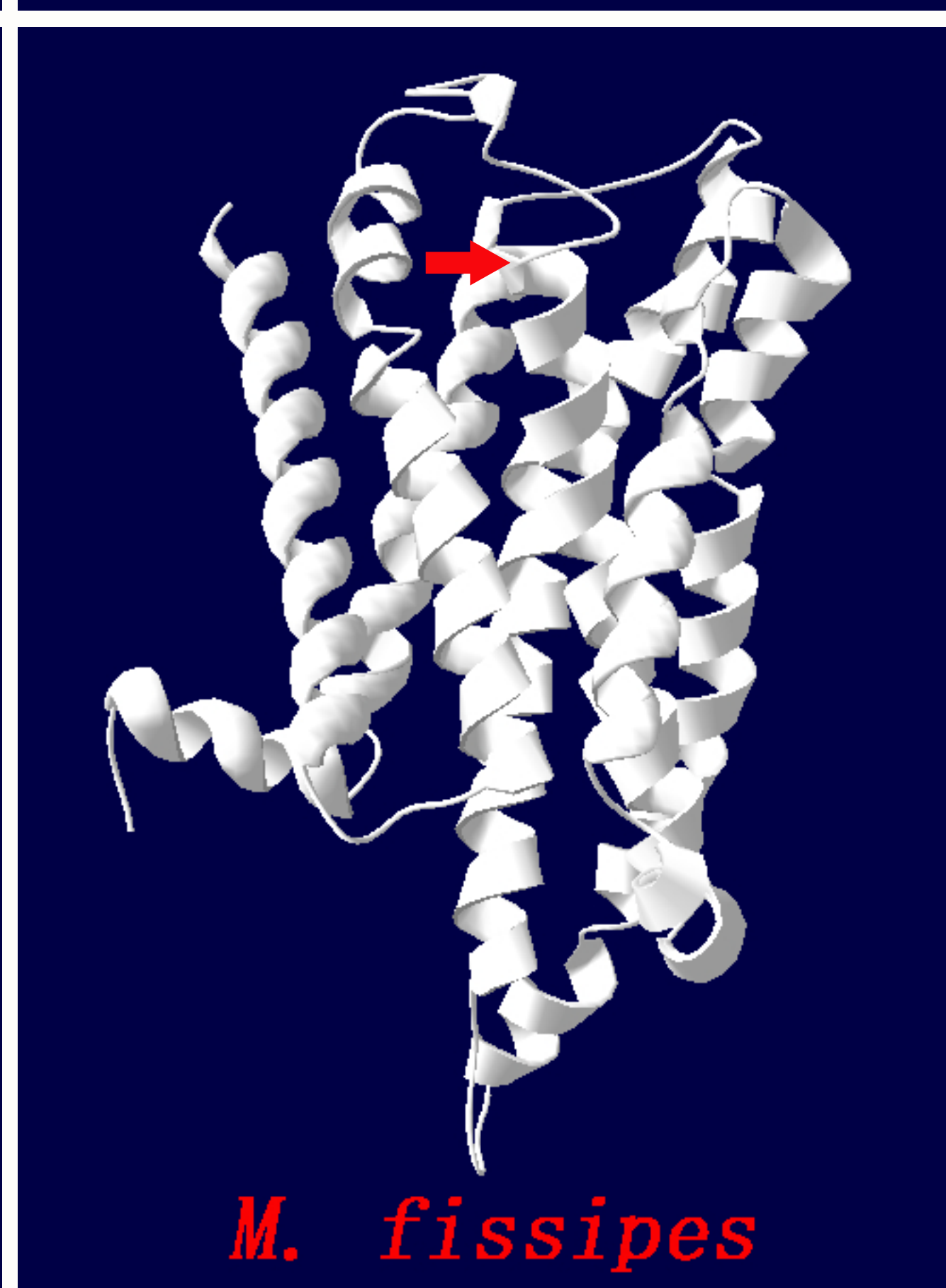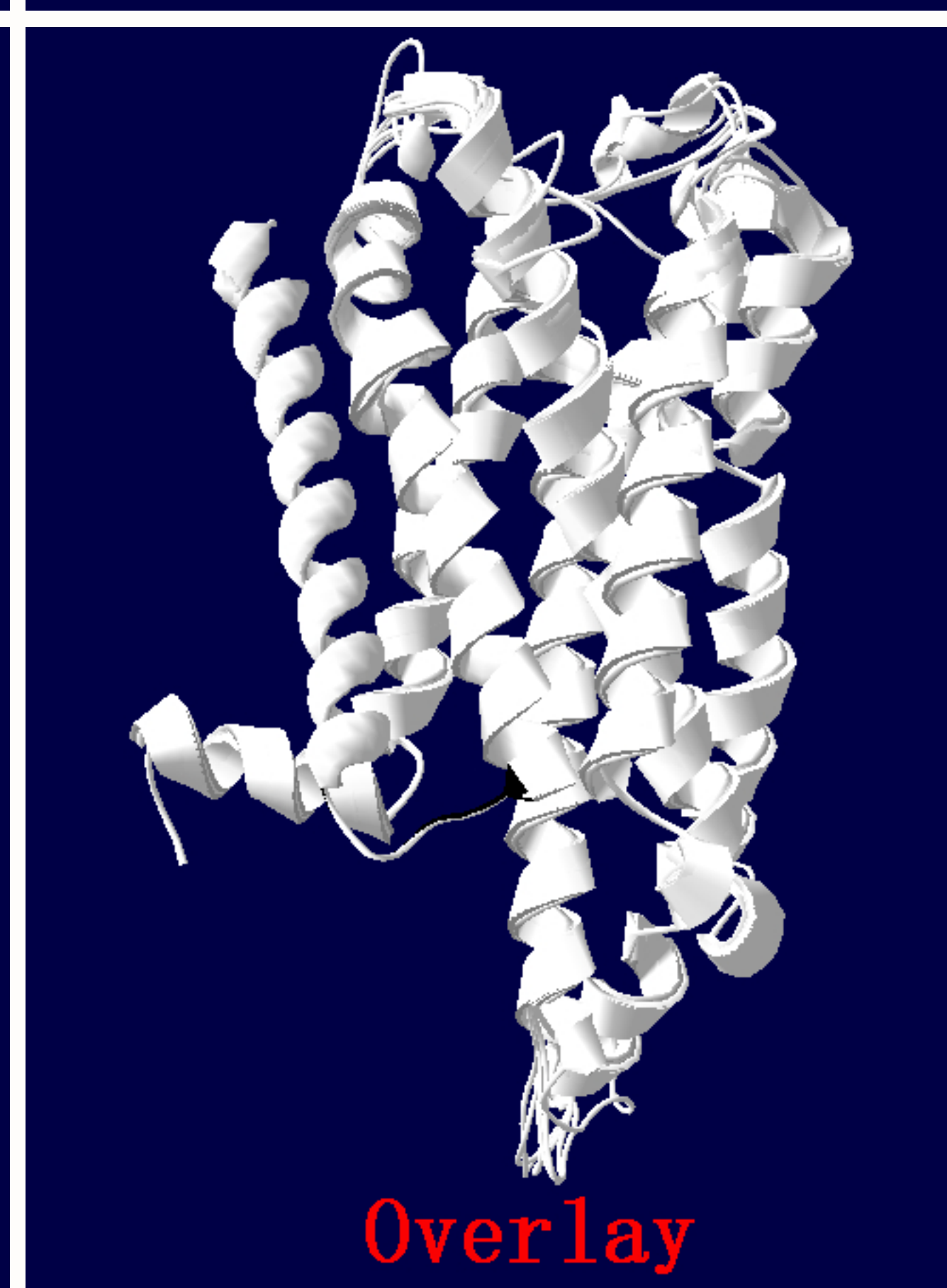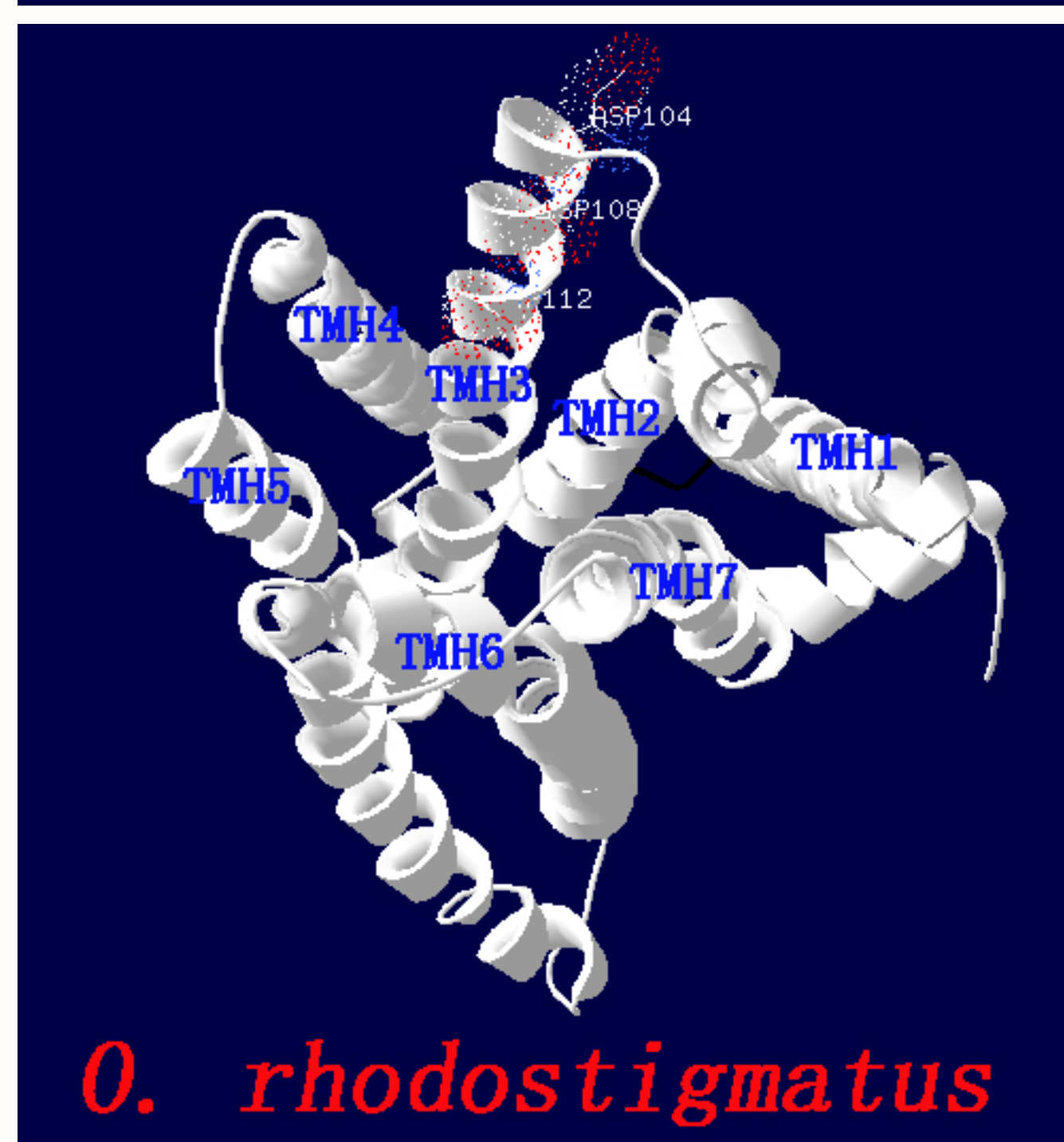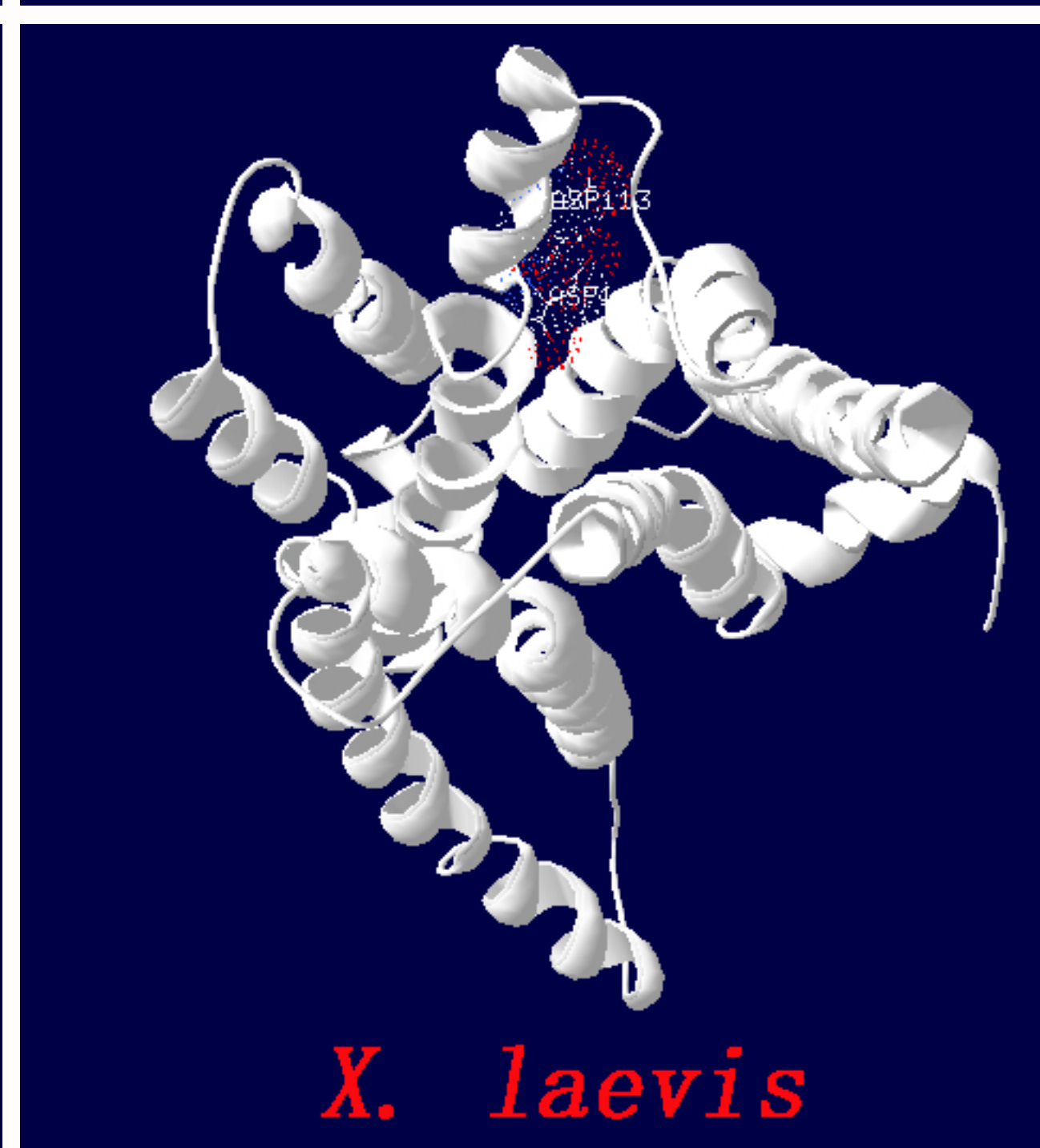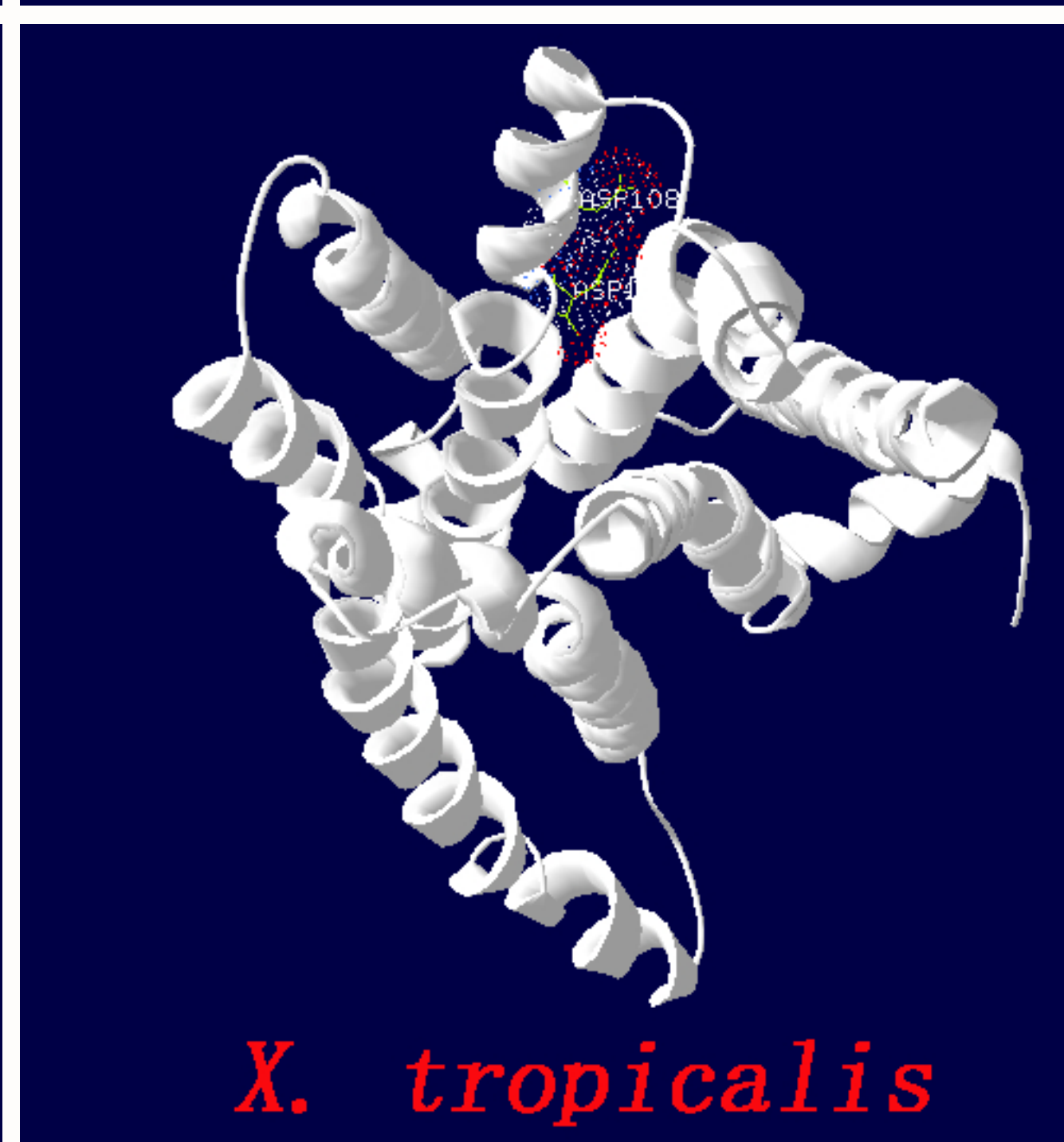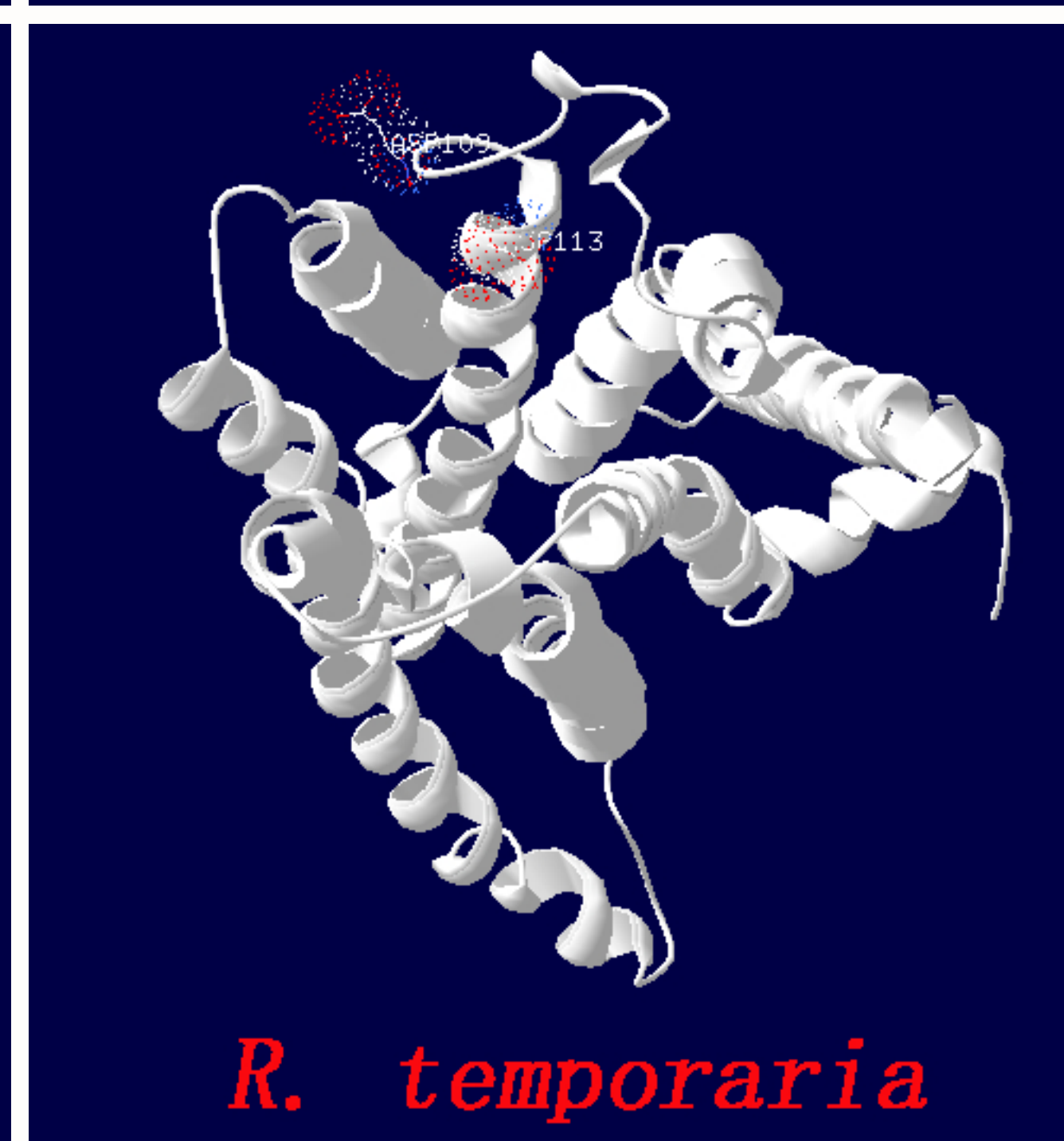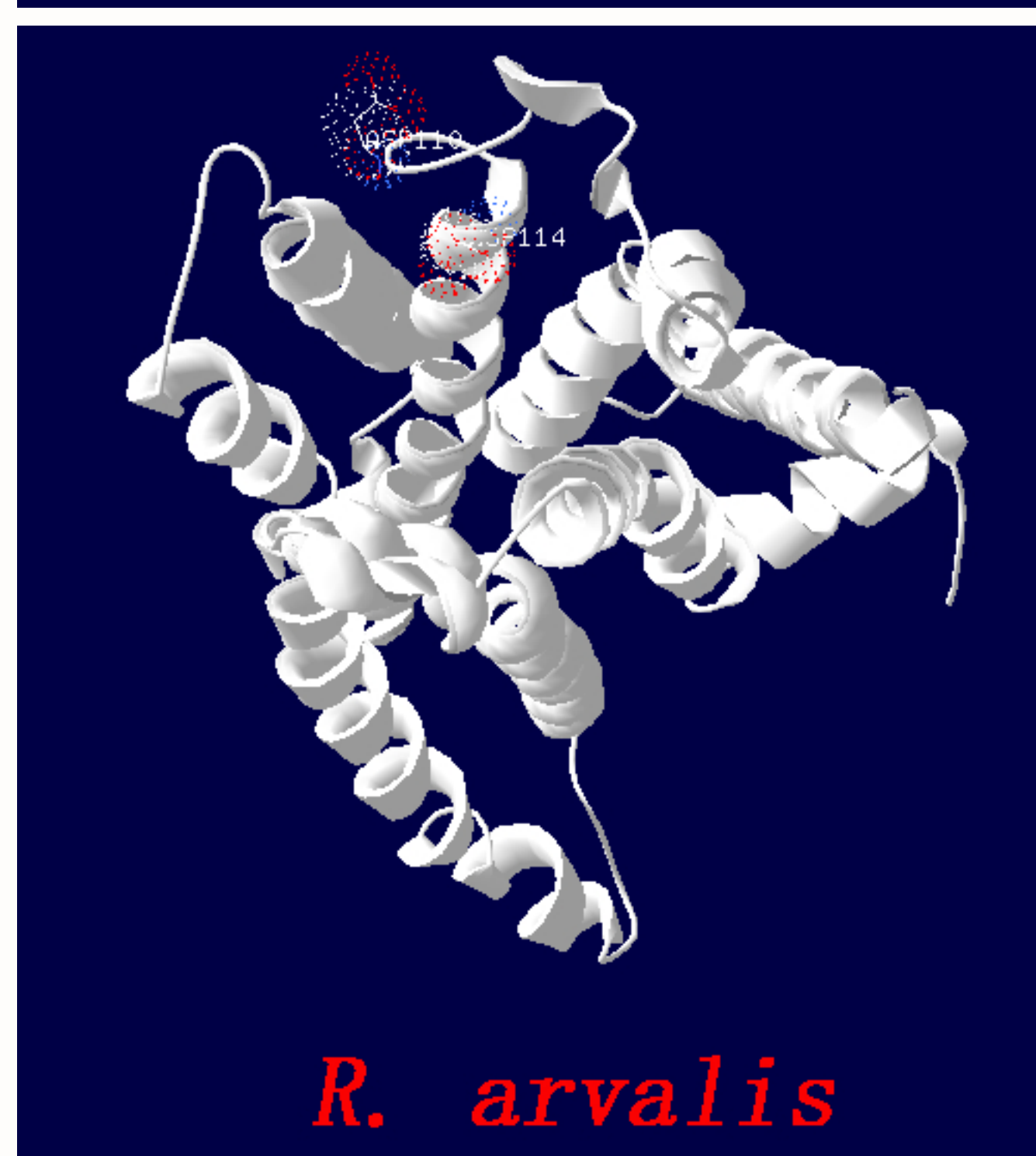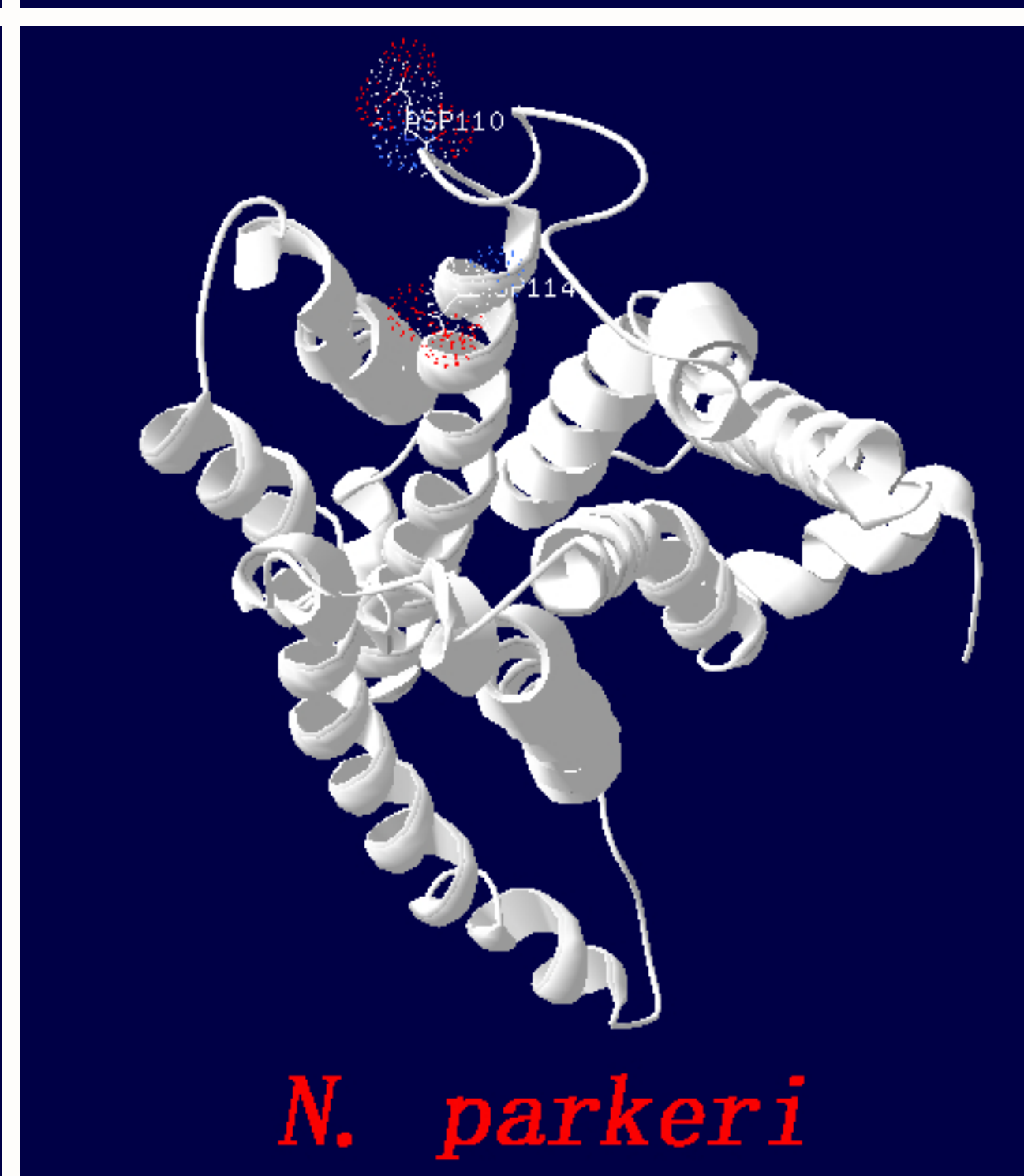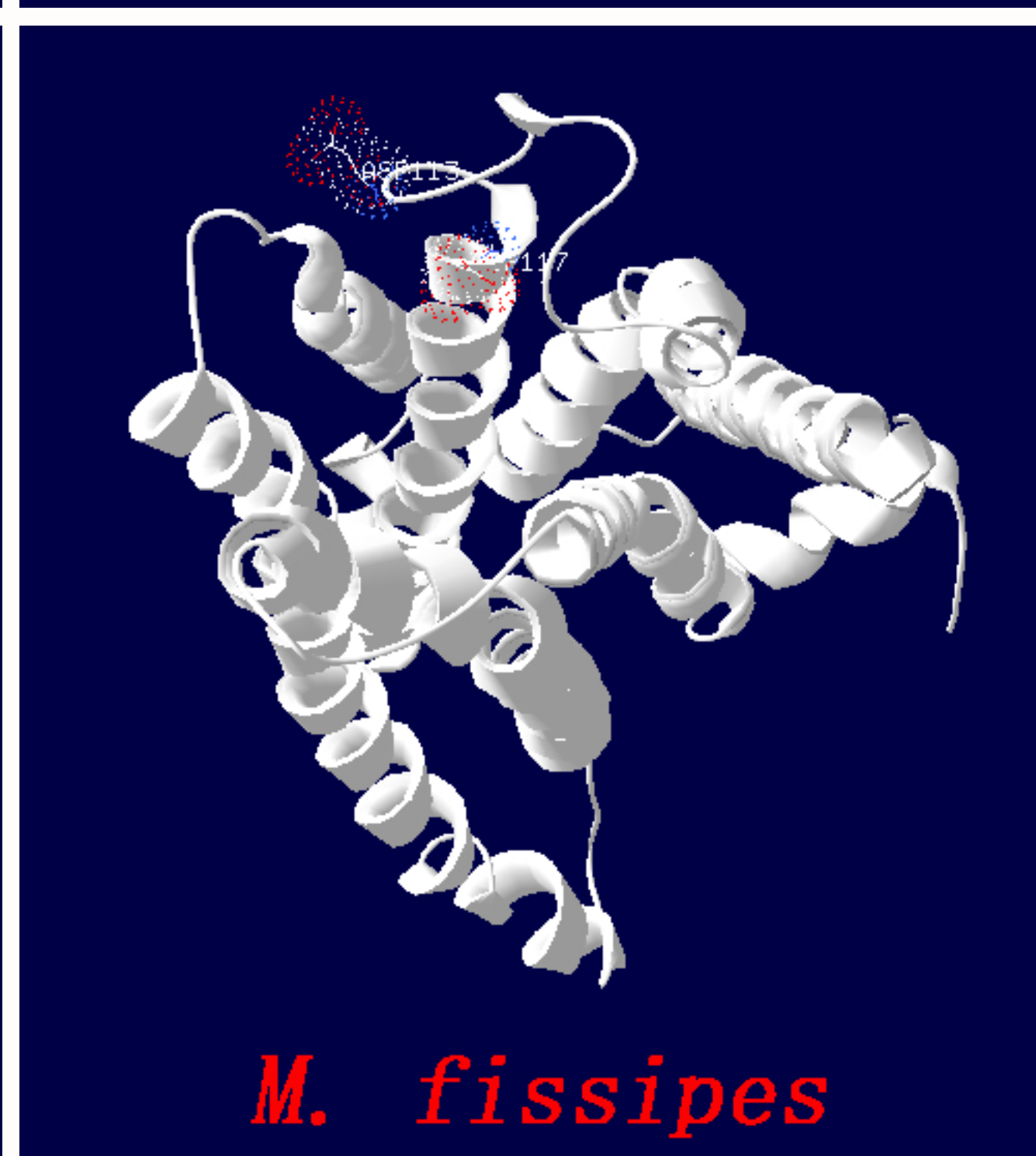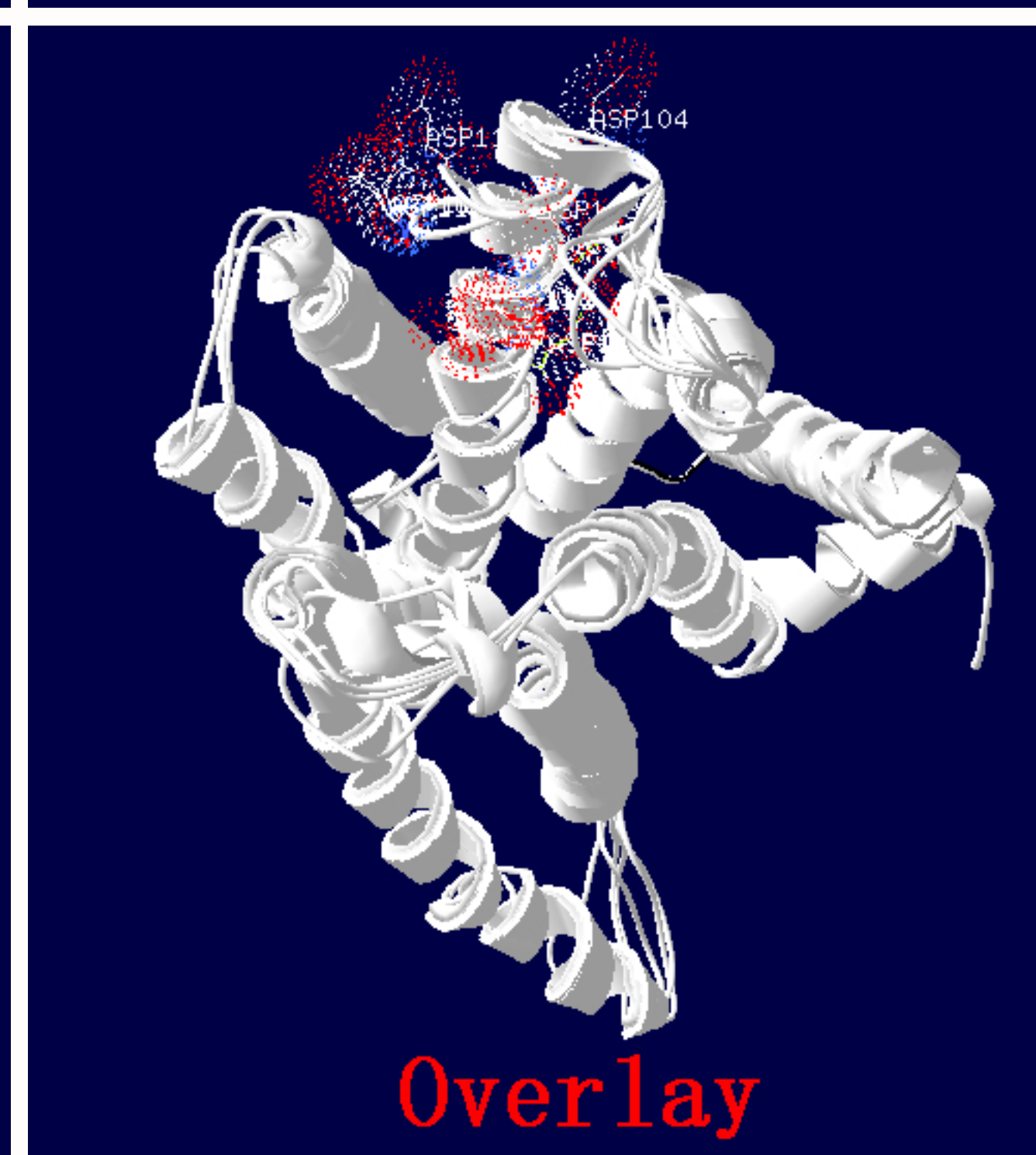

Supplement: Supplementary file 7 — Figure S2. 3D-model of MC1R in frogs. Models are built based on human 5-hydroxytryptamine receptor. Red arrows indicate ending point of TMH3 in extracellular side. (PDF 11742 kb) [file 12864_2018_4790_MOESM7_ESM.pdf]
